# Graphene‐Coupled Terahertz Semiconductor Lasers for Enhanced Passive Frequency Comb Operation

**DOI:** 10.1002/advs.201900460

**Published:** 2019-08-23

**Authors:** Hua Li, Ming Yan, Wenjian Wan, Tao Zhou, Kang Zhou, Ziping Li, Juncheng Cao, Qiang Yu, Kai Zhang, Min Li, Junyi Nan, Boqu He, Heping Zeng

**Affiliations:** ^1^ Key Laboratory of Terahertz Solid State Technology Shanghai Institute of Microsystem and Information Technology Chinese Academy of Sciences 865 Changning Road Shanghai 200050 China; ^2^ Center of Materials Science and Optoelectronics Engineering University of Chinese Academy of Sciences Beijing 100049 China; ^3^ State Key Laboratory of Precision Spectroscopy East China Normal University Shanghai 200062 China; ^4^ i‐Lab Suzhou Institute of Nano‐Tech and Nano‐Bionics Chinese Academy of Sciences 398 Ruoshui Road Jiangsu 215123 Suzhou China; ^5^ School of Optical Electrical and Computer Engineering University of Shanghai for Science and Technology Shanghai 200093 China

**Keywords:** frequency combs, on‐chip dual‐combs, pulse generation, terahertz lasers

## Abstract

Optical frequency combs, consisting of well‐controlled equidistant frequency lines, have been widely used in precision spectroscopy and metrology. Terahertz combs have been realized in quantum cascade lasers (QCLs) by employing either an active mode‐locking or phase seeding technique, or a dispersion compensator mirror. However, it remains a challenge to achieve the passive comb formation in terahertz semiconductor lasers due to the insufficient nonlinearities of conventional saturable absorbers. Here, a passive terahertz frequency comb is demonstrated by coupling a multilayer graphene sample into a QCL compound cavity. The terahertz modes are self‐stabilized with intermode beat note linewidths down to a record of 700 Hz and the comb operation of graphene‐coupled QCLs is validated by on‐chip dual‐comb measurements. Furthermore, the optical pulse emitted from the graphene‐coupled QCL is directly measured employing a terahertz pump–probe technique. The enhanced passive frequency comb operation is attributed to the saturable absorption behavior of the graphene‐integrated saturable absorber mirror, as well as the dispersion compensation introduced by the graphene sample. The results provide a conceptually different graphene‐based approach for passive comb formation in terahertz QCLs, opening up intriguing opportunities for fast and high‐precision terahertz spectroscopy and nonlinear photonics.

## Graphical abstract

**Enhanced passive frequency comb operation and pulse generation in terahertz semiconductor lasers, i.e., quantum cascade lasers**, are successfully demonstrated by coupling a multilayer graphene saturable absorber into a 6 mm long terahertz laser. Dual‐comb and pump–probe techniques are implemented to verify the frequency comb and pulsed operations (16 ps optical pulses) of graphene‐coupled terahertz lasers.

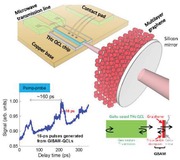

Frequency combs^1^, ^2^ have been successfully used in a range of important applications such as absolute optical‐frequency measurements,^3^ optical clocks,^4^ and long‐baseline interferometry.^5^ Inspired by this, broadband frequency combs in the mid‐infrared and terahertz regimes have been put to use in promising applications such as high‐resolution spectroscopy^6^ and high‐sensitivity terahertz near‐field microscopy.^7^ Electrically pumped terahertz quantum cascade lasers (QCLs)^8^, ^9^ with the advantages of broadband frequency coverage,^10^, ^11^, ^12^ low beam divergence,^13^ and high power^14^, ^15^ are ideal candidates for controlled terahertz radiation. In particular, frequency modes of a QCL can be coherently locked in phase by using active mode‐locking^16^, ^17^, ^18^ or seeding techniques.^19^, ^20^, ^21^, ^22^ Besides the active tools, the comb operation in free‐running QCLs has been also obtained by compensating the cavity dispersions using Gires–Tournois interferometer (GTI) or double‐chirped mirrors.^23^, ^24^, ^25^ However, most of the active mode‐locking and GTI mirrors involve either complex microwave electronics, or delicate mirror designs and complicated fabrication processes. These techniques could be simplified by employing a passive mechanism to reduce phase noise and self‐stabilize comb modes.

The general scheme employed for passive comb formation is to get a laser mode‐locked by incorporating saturable absorbers or other nonlinear reflectors in the laser cavity.^26^, ^27^, ^28^, ^29^ This technique functions well in interband diode lasers and fiber lasers in which doped quantum well structures, graphene,^30^ or other saturable absorbers are widely used. However, for QCLs, although the modulation of light has been demonstrated by using quantum well or graphene absorbers,^31^, ^32^ saturable absorber‐based passive comb formation and mode‐locking have so far not been achieved.^16^ Actually, in terahertz QCLs, the gain recovery time was measured to be in the range of 10–50 ps depending on the active region design and operation condition of the lasers,^33^, ^34^, ^35^, ^36^ which can in principle support passive comb formation or even mode‐locking. However, due to the insufficient intracavity nonlinear absorption, it remains a great challenge to self‐stabilize comb modes with ultralow phase noise or ultranarrow linewidths. In this work, we construct a QCL compound cavity with a terahertz nonlinear reflector based on a multilayer graphene sample, demonstrating enhanced passive comb operation and pulse generation in terahertz QCLs.

**Figure** **1**a shows the schematic of our graphene‐coupled terahertz semiconductor laser. The 15‐layer graphene sample that is transferred onto a 0.3 mm thick silicon (Si) mirror is placed close to the as‐cleaved laser facet to form the compound cavity. The inset of Figure 1a illustrates the compound laser cavity geometry and the terahertz light propagation. To enhance the terahertz feedback, we use a single plasmon waveguide terahertz laser with a far‐field beam divergence of ≈20° in both the horizontal and vertical directions (see Figure S3 in the Supporting Information). The microwave transmission line positioned behind the laser chip is used for direct current (DC) injection, intermode beat note, and dual‐comb measurements. Note that by coupling the graphene into the QCL compound cavity, the air gap and the air/Si interface construct a GTI mirror, which has been widely used to compensate the spectral dispersion of laser gain mediums for achieving comb operation.^23^, ^25^, ^37^, ^38^, ^39^ In Figure S5 in the Supporting Information, we evaluate dispersion effects arisen from the specific GTI mirror constructed in our compound cavity. Furthermore, the compensation effect introduced by the graphene sample and its contribution to the laser passive comb operation will be discussed in detail in the main paper.

**Figure 1:**
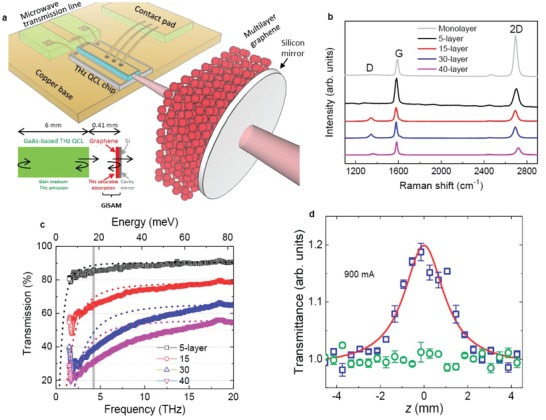
Schematic of the graphene‐coupled QCL and characterizations of its constituent graphene absorbers. a) 3D view of the laser configuration. The 0.3 mm thick Si acts as an optical feedback mirror and serves as a holder for the multilayer graphene. Inset: Illustration of the terahertz light propagation in the compound cavity. The 0.41 mm long air gap and the left air/Si interface construct an extended cavity to the QCL cavity. The extended cavity also acts as a GTI mirror. The QCL ridge is 6 mm long and 150 µm wide. b) Raman spectra of the graphene films. c) Transmission spectra of the multilayer graphene films in the terahertz frequency range. The vertical gray band shows the frequency at ≈4.2 THz, corresponding to the emission frequency of the terahertz QCL used in this work (see Figure 3c; Figure S15, Supporting Information). The fitting parameters used for the dashed lines are *E*_f_ = 158 meV and τ ≈ 500 fs. d) Normalized transmission of a 15‐layer graphene saturable absorber (squares) and Si substrate (circles) measured using an open‐aperture *z*‐scan technique as the GiSAM‐QCL is operated at 900 mA.

In this work, a relatively long laser cavity (6 mm) is intentionally used to obtain an intermode beat note frequency of ≈6 GHz (see **Figure** **2**). Compared to short‐cavity lasers, the 6 mm long laser shows a significant improvement in the terahertz frequency stability due to the stronger locking mechanism induced by the four‐wave mixing in the cavity. Such improvement is experimentally verified by intermode beat note measurements (see Figure 2 and the Supporting Information). The light–current–voltage characteristics of the 6 mm long and 150 µm wide terahertz QCL is depicted in Figure S1b in the Supporting Information. The measured maximum terahertz power in continuous‐wave (CW) mode is ≈2 mW at 15 K.

**Figure 2:**
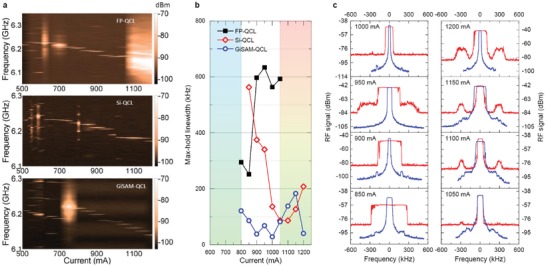
Characterization of mode coherence of the terahertz lasers using an intermode beat note technique. a) Evolution of the intermode beat note frequency as a function of drive current for the FP‐QCL (upper panel), Si‐QCL (middle panel), and GiSAM‐QCL (lower panel). All beat note traces are recorded with a resolution bandwidth of 100 kHz and 20‐time average. b) Measured “max‐hold” linewidth as a function of drive current for FP‐QCL (squares), Si‐QCL (diamonds), and GiSAM‐QCL (circles). c) Intermode beat note spectra in the “max‐hold” mode of FP‐QCL (red line) and GiSAM‐QCL (blue line) for current ranging from 850 to 1200 mA. In (c), the central frequency of each spectrum around 6.2 GHz is subtracted for clear comparison. All experimental data are taken with the laser operating in CW mode at a temperature of 15 K.

The multilayer graphene films are fabricated by transferring monolayer graphene onto the Si mirror one by one layer to maximally decouple the neighboring graphene monolayers.^40^ This is to ensure that the absorber is graphene‐like rather than graphite‐like (see the Experimental Section for details of the fabrication procedures). The topography of the fabricated graphene films with different layers measured by atomic force microscopy (AFM) shows that the number of graphene layers can be well controlled (see Figure S1d in the Supporting Information). The Raman spectroscopy (Figure 1b) demonstrates the high quality of the graphene films, which is verified by the sharp G and 2D bands and ultralow D band.

Both linear and nonlinear absorptions are critical for the functioning of graphene saturable absorbers. A moderate linear absorption loss can shorten the optical pulses for passive mode‐locking. However, if the linear absorption introduced by graphene absorbers is too high, the saturable absorption would hardly happen and optical pulses cannot be obtained. The linear transmission of the multilayer graphene samples in terahertz frequency range, as shown in Figure 1c, is measured by a Fourier transform infrared spectrometer. Around the operation frequency (4.2 THz) of the terahertz laser, as marked by the vertical line in Figure 1c, the transmissions of the 5‐, 15‐, 30‐, and 40‐layer graphene samples are measured to be 86%, 66%, 38%, and 31%, respectively. Note that fabrication imperfections during layer‐by‐layer transfer of monolayer graphene (see the Experimental Section) and the interfacial dangling bonds of the integrated graphene monolayers can bring about additional absorptions. As a consequence, we observe a fast decrease in transmission as the number of graphene layers increases. In Figure 1c, we also display theoretical fits to the transmission curves in order to better understand the terahertz absorption processes in the multilayer graphene samples. The fits are performed based on the Drude intraband model,^41^ with the fitting parameters being momentum scattering time τ and Fermi energy *E*_f_ (see the Supporting Information). Since the terahertz photon energy is much smaller than the Fermi energy of 158 meV (see Figure S8 in the Supporting Information), the terahertz absorption in the graphene samples is an intraband transition process. Because the fabrication imperfection is accumulated during the layer‐by‐layer transferring process, the fitting curves show larger deviations from the experimental results for 30‐ and 40‐layer graphene samples. To make the graphene absorber function properly, we use a 15‐layer graphene sample in the following experiment to provide moderate linear absorption loss and sufficient nonlinearity.

The nonlinear absorption behavior of the graphene sample is investigated using a *z*‐scan experiment which has been used to characterize the saturable absorption of nonlinear materials.^42^ The open‐aperture *z*‐scan technique is fully described in the Experimental Section and the Supporting Information. Figure 1d plots the transmission of a 15‐layer graphene (squares) measured when the graphene‐coupled QCL is operated at 900 mA in CW, which is normalized by the transmission of the Si substrate (circles). A saturable absorption signature (increase in transmission by 20% at the focal point *z*_0_) can be clearly observed. A theoretical fit is performed to extract the saturation parameters of the graphene sample. The average power of the QCL with a graphene‐integrated saturable absorber mirror (GiSAM) measured at *z*_0_ at 900 mA is 685 µW, which corresponds to a power density *I*_0_ = 2 W cm^−2^ by considering a beam waist diameter of 209 µm at *z*_0_ (see Figure S9 and Video S1 in the Supporting Information). The fit (red curve in Figure 1d) yields a saturation intensity *I*_s_ = 0.73 ± 0.12 W cm^−2^, a nonsaturable absorption coefficient α_NS_ = 0.10 ± 0.03, and a saturable absorption coefficient α_S_ = 0.21 ± 0.01. If we assume an optical pulse width of 16 ps or a duty cycle of 10% (see Figure 4), the peak power density and saturation peak intensity are supposed to be 20 and 7.3 W cm^−2^, respectively. All these saturation parameters are obtained based on measurements for the graphene sample outside the QCL cryostat. We also evaluate the peak power density and pulse energy on the GiSAM in the QCL compound cavity. By taking into account all the losses introduced by water absorption, mirror reflection, window transmission, etc. (see Supporting Information), an average power of 2 mW measured outside the cryostat is equivalent to 10 mW on the GiSAM in the cryostat. This can then be translated into a power density of 61 W cm^−2^ by considering the beam divergence and the air gap between the laser facet and the GiSAM. Finally, one can estimate a peak intensity of 610 W cm^−2^ or a pulse energy of 9.7 nJ cm^−2^. The estimated peak intensity of 610 W cm^−2^ is much larger than *I*_s_ derived from the fit in Figure 1d, which indicates that the terahertz power is far sufficient to saturate the graphene sample in the compound cavity. It is worth noting that to avoid thermal issues in the *z*‐scan experiment, normally the terahertz laser should be driven in pulsed mode or it is optically pulsing. However, the saturable absorption was also observed when terahertz QCLs operated in a quasi‐CW mode (with a duty cycle of 40%).^42^ This is possibly due to that a high repetition rate and short pulse width are applied and therefore the thermal effect can be reduced, thanks to the fast carrier dynamics of the graphene sample. In Figure S12 in the Supporting Information, the normalized transmission of the graphene sample as a function of average power of the GiSAM‐QCL for various *z* positions is shown. The nonlinear behavior of the graphene sample is related to the carrier absorption process. Once the absorption is fully saturated, the graphene will be transparent to the terahertz light. The nonlinear increase in transmission can be only observed before the graphene is fully saturated and afterward the power dependence of the transmission is linear. Since the saturation regime is easily realized in our experiment especially for *z* = 0, we are not able to observe the clear nonlinear increase in transmission with power. However, for *z* = −1 and 1 mm, as the threshold average power for saturable absorption is higher than the case of *z* = 0, the nonlinear behavior is observed as shown in Figure S12 in the Supporting Information.

Moreover, further experiments are implemented to investigate the saturable absorption of the graphene sample. In Figure S2 in the Supporting Information, the light–current curves for the QCL with Si mirror and GiSAM measured in the constant current mode are shown. We can see that the slope efficiency changes with increasing the current for the QCL with GiSAM, while it remains constant in the entire current dynamic range for the QCL with a Si mirror. This infers the nonlinearity introduced by the graphene sample. To verify that the saturable absorption observed in Figure 1d is not due to the nonuniformity of the graphene sample, we show another two *z*‐scan traces measured at 900 mA at different spots of the sample (see Figure S11 in the Supporting Information). As expected, all the *z*‐scan traces measured with the GiSAM‐QCL at 900 mA show almost identical behavior, which confirms that the graphene sample used in this work is uniform and the nonlinear saturable absorption is obtained when the sample is illuminated by the GiSAM‐QCL.

The mode stability of GiSAM‐QCLs is first studied employing an electrical intermode beat note measurement (see the Experimental Section) which has been widely used to characterize the mode coherence of terahertz lasers by recording the down‐converted beating signal of optical modes.^17^, ^18^, ^43^ Figure 2 shows the comparison of intermode beat note linewidths measured from the 6 mm QCLs with different cavity configurations, i.e., Fabry–Pérot single‐cavity QCL (FP‐QCL), linear compound‐cavity QCL with a Si mirror (Si‐QCL), and nonlinear compound‐cavity QCL with the GiSAM (GiSAM‐QCL). The significant linewidth reduction by coupling the GiSAM is clearly observed in Figure 2a even by naked eyes.

We further monitor the long‐term frequency stability of the QCL modes by switching on the “max‐hold” function of the spectrum analyzer to record and store the spectral maxima during the time duration (3 min) of the measurement. Figure 2b summarizes the measured “max‐hold” linewidths of the intermode beat notes as a function of drive current. The “max‐hold” linewidth drops from 600 kHz for free‐running FP‐QCLs to 30 kHz for GiSAM‐QCLs operating in the drive current range between 900 and 1000 mA. In addition, we can clearly see that GiSAM‐QCLs exhibit narrower “max‐hold” linewidths than the linear compound cavity Si‐QCLs. At most drive currents between 800 and 1200 mA, GiSAM‐QCLs exhibit “max‐hold” linewidths less than 100 kHz, while Si‐QCLs may attain comparable “max‐hold” linewidths at drive currents larger than 1000 mA, but broad spectral sidebands appear to compete with central modes (Figure 2c). The observed reduction in “max‐hold” linewidth clearly indicates that the GiSAM‐based nonlinear compound cavity benefits self‐stabilization of QCL modes. It is worth noting that in single‐shot linewidth measurements, GiSAM‐QCLs exhibit a typical linewidth in the level of hundred Hertz as shown in **Figure** **3**b.

**Figure 3:**
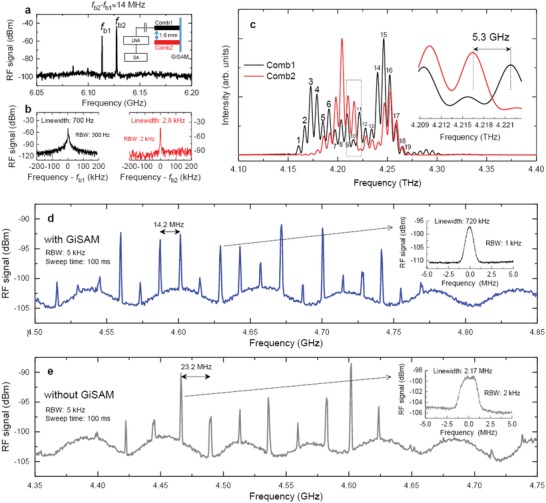
On‐chip dual‐comb. a) Intermode beat note spectra of the dual‐comb device. *f*_b1_ and *f*_b2_ denote the beat note frequencies of GiSAM‐QCL Comb1 and Comb2, respectively. The difference of these two beat note frequencies is measured to be ≈14 MHz. The inset of (a) depicts the experimental setup of the dual‐comb measurement. The two laser combs have the identical dimensions, i.e., 150 µm wide and 6 mm long. LNA: low noise amplifier; SA: spectrum analyzer. b) Linewidths of *f*_b1_ (left panel) and *f*_b2_ (right panel). c) Terahertz emission spectra of GiSAM‐QCL Comb1 (black) and Comb2 (red). The inset shows the zoom‐in of the spectrum outlined in the rectangle. d) GiSAM‐QCL dual‐comb spectrum measured with a resolution bandwidth (RBW) of 5 kHz and a sweep time of 100 ms. The typical mode linewidth is depicted in the inset. For the measurement, the GiSAM‐QCL Comb1 is pumped at a current of 975 mA and Comb2 is pumped at a current of 1029 mA. The heat sink temperature is stabilized at 29.5 K. e) Dual‐comb spectrum of FP‐QCLs without GiSAM. The inset shows the typical mode linewidth. The nonflat background in (d) and (e) is resulted from the LNA.

Although the intermode beat note of the GiSAM‐QCL demonstrates narrow linewidth and high stability, it is an indirect method for frequency comb characterization since it cannot prove that all terahertz modes contribute to the narrow intermode beat note signal. Therefore, one needs to perform further experiments, such as intermode beat note interferogram^23^, ^44^ or dual‐comb spectroscopy^6^, ^45^ to fully prove the broadband frequency comb operation. Figure 3 summarizes the experimental results of the on‐chip dual‐comb spectroscopy using GiSAM‐QCLs and FP‐QCLs. The two laser combs have identical ridge dimensions, 150 µm wide and 6 mm long, which are the same as the aforementioned laser in Figure 1. Due to the mode coupling, the intermode beat note and dual‐comb spectra are measured using Comb1 as a fast detector. The intermode beat notes of the two GiSAM‐QCL combs are shown in Figure 3a. When the two laser combs are electrically pumped at different currents (975 mA for Comb1 and 1029 mA for Comb2), the measured intermode beat note signals, *f*_b1_ and *f*_b2_, are separated by 14 MHz. Here, we intentionally separate the two laser combs by 1.6 mm (inset of Figure 3a) to avoid an optical injection locking of the two lasers. The linewidths of two intermode beat notes are measured to be 700 Hz and 2.8 kHz for Comb1 and Comb2, respectively, as shown in Figure 3b.

Figure 3c plots the terahertz emission spectra in linear scale of the two GiSAM‐QCL combs (black lines for Comb1 and red lines for Comb2). The emission spectrum of one laser comb is measured when the other laser is switched off. We can see that the spectra of two laser combs are strongly overlapped in the frequency range between 4.15 and 4.3 THz. We mark the modes with index numbers from 1 to 19 for Comb1, which are supposed to beat with the modes of Comb2 and then contribute to the down‐converted dual‐comb spectrum. Figure 3d displays all the 19 down‐converted frequency lines equally spaced by 14.2 MHz that is consistent with *f*_b2_–*f*_b1_ (see Figure 3a). The central frequency of the down‐converted dual‐comb lines, accurately determined by beating of two close modes of Comb1 and Comb2, is 4.65 GHz. This, however, shows a discrepancy with the frequency difference of 5.3 GHz as shown in Figure 3c due to a low spectral resolution of 0.1 cm^−1^ (3 GHz) of the Fourier transform infrared spectrometer. The down‐converted dual‐comb spectrum shown in Figure 3d indicates that at least 19 equally spaced terahertz modes in each laser comb are coherent, and they contribute to the narrow intermode beat note. In the inset of Figure 3d, we show the typical linewidth of 720 kHz for one dual‐comb mode. As expected, the linewidth of the dual‐comb line is much wider than the intermode beat note linewidth shown in Figure 3b because the comb carrier offset noise also contributes to the linewidth broadening of dual‐comb lines.

We next compare the dual‐comb spectrum of FP‐QCLs with that of GiSAM‐QCLs. Similarly with Figure 3a–c, we measure the intermode beat note and terahertz emission spectra of the two FP‐QCLs (see Figures S17–S19 in the Supporting Information). Figure 3e presents the dual‐comb spectrum of the FP‐QCLs with the GiSAM removed. We do observe significant differences between Figure 3d and Figure 3e. First of all, the intermode beat note linewidths of GiSAM‐QCLs are much narrower than those of FP‐QCLs, i.e., 700 Hz (GiSAM‐QCL Comb1) versus 240 kHz (FP‐QCL Comb1). In addition, in the down‐converted dual‐comb spectrum of the FP‐QCLs, we only observe 11 modes and a typical linewidth is measured to be 2.17 MHz, while the number of modes and typical linewidth of the GiSAM‐QCL dual‐comb spectrum are 19 and 720 kHz (see Figure 3d), respectively. Although at least 18 terahertz modes of each FP‐QCL comb overlap with each other (see Figure S19 in the Supporting Information), which are similar to the terahertz spectra of GiSAM‐QCLs, the mode decoherence results in only 11 dual‐comb lines. It means that a part of terahertz modes are not stable in the free‐running FP‐QCLs and these modes cannot contribute to the comb operation. The number of dual‐comb lines can be translated into the optical comb bandwidth, i.e., 110 and 61 GHz bandwidths for GiSAM‐QCLs and FP‐QCLs, respectively, by considering the mode spacing shown in Figure 3a. From the comparison, we can conclude that the GiSAM does contribute to the enhanced passive comb operation in the GiSAM‐QCL.

Finally, to further prove that the GiSAM‐QCL is pulsing, we perform a terahertz pump–probe experiment. **Figure** **4**a shows the experimental setup that employs the GiSAM‐QCL as the comb source and a 15‐layer graphene sample as a saturable absorber. The terahertz pulses emitted from the GiSAM‐QCL comb are split into two beams, one is the pump beam which is used to pump the 15‐layer graphene sample to achieve the saturable absorption (see Figure 1d), while the other is the probe beam which is crossly overlapped with the pump beam on the graphene sample. A delay line is added into the probe beam to sample the terahertz pulses. After transmission, the two beams are spatially separated, which facilitates the detection of the change in transmission of the probe beam. Figure 4b depicts the transmission of the probe beam as a function of time delay. We observe two peaks separated by 160 ps, corresponding to the inverse of the repetition rate of the terahertz pulses. The terahertz pulse width is measured to be 16 ps as shown in Figure 4b, which directly confirms the pulse generation in the GiSAM‐QCL.

**Figure 4:**
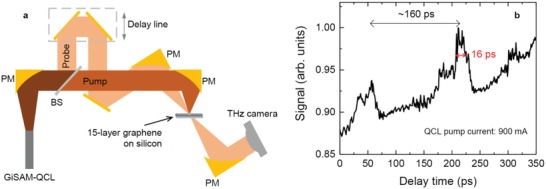
Terahertz pump–probe. a) Experimental setup of the terahertz pump–probe employing the GiSAM‐QCL comb source. The transmitted (pump) beam is used for pumping the graphene sample to achieve saturable absorption, and the reflected (probe) beam is collimated onto the same position of the graphene sample as the pump beam but propagating toward a different direction for detection. PM: parabolic mirror; BS: beam splitter. b) Measured transmitted light intensity of the probe beam as a function of delay time. The QCL is pumped at 900 mA at 15 K. The increasing baseline in (b) is due to the thermal issue of the terahertz camera.

The experimental data shown in Figures 2, 3, 4 indicate that the graphene does take effect for the enhanced passive comb formation and pulse generation in terahertz QCLs. In the following, we discuss the possible origins of the enhanced comb operation. First of all, as shown in Figure 1d, the saturable absorption of the graphene sample that results in the passive mode‐locking can be a reason for the pulse generation in GiSAM‐QCLs. Although a recent experiment employing a pump–probe spectroscopy shows that the gain recovery time of a terahertz QCL with the hybrid active region design can reach 50 ps,^36^ it is still far less than the laser round trip time ≈160 ps in the current laser. Since it is hard to quantify the gain recovery time of the terahertz QCL emitting at high frequency (>4 THz) used in this work, the passive mode‐locking resulted from the graphene saturable absorption cannot be fully verified and therefore we can only say that the saturable absorption is a possible origin of the enhanced comb operation in the GiSAM‐QCL. Even though, here we want to mention another important parameter for the passive mode‐locking based on saturable absorbers, i.e., carrier recovery time in graphene. Although the passive mode‐locking of lasers is not determined by whether a saturable absorber is fast or slow, the carrier recovery time of the absorber is important for the pump–probe measurement shown in Figure 4. To give a complete description of the multilayer graphene absorber, we summarize the carrier dynamics of graphene in terahertz frequency range as follows. At room temperature, under a terahertz pump, the measured recovery time of graphene is less than 1 ps.^46^ Since the carrier recovery time is significantly smaller than the measured pulse duration shown in Figure 4b, the pump–probe experiment finally results in the measurement of the optical pulses of the GiSAM‐QCL. At low temperature, the carriers in graphene relax much slower. A terahertz pump–probe measurement using a free‐electron laser as a source for tunable picosecond pulses of infrared radiation reveals that the carrier recovery time at 10 K can be hundreds of picoseconds.^41^

The other origin of the enhanced passive frequency comb operation in the GiSAM‐QCL is the dispersion compensation introduced by coupling the graphene sample that slightly changes the reflectivity of the GTI mirror. Theoretical studies show that the graphene is not able to significantly change the terahertz reflectivity at the air/Si interface^47^ and therefore the dispersion introduced by graphene should be very limited. Even though, an effect of dispersion by coupling graphene is evaluated by considering various reflectivity changes (from −10% to 10%) induced by graphene as shown in Figure S5d in the Supporting Information. We can see that when a reduction in reflectivity is introduced by the graphene sample, the total dispersion of the laser can be essentially compensated, which in principle favors the passive frequency comb operation of the GiSAM‐QCL.

Compared with existing methods such as active mode‐locking or using passive mirrors to compensate dispersions, the passive way of using a graphene absorber to achieve frequency comb operation shows two advantages/merits. First of all, the passive technique in this work can get rid of complex electronics and optics for intracavity loss modulation and active mode stabilization, and/or mirror designs and fabrications for compensating the group velocity dispersions in QCLs. Furthermore, the microwave impedance mismatching is not necessary to be taken into account in the passive scheme employed in this work. Thus, the passive comb system is more compact than the active terahertz QCL combs. Second, the passive scheme (in principle) allows the generation of much shorter pulses than the active one because a saturable absorber can modulate the losses much faster than electronic modulators. However, this doesn't mean that the absorber should be fast to achieve passive mode‐locking. We only require the absorber to respond fast to the leading wing of the pulse. Sometimes, a slow absorber is even better for achieving self‐starting mode‐locking in lasers.^48^ The reported pulse durations from actively mode‐locked terahertz QCLs are in a level of few picoseconds.^18^, ^25^, ^49^ Currently, the 16 ps pulse duration from this work is still longer than those obtained from actively mode‐locked lasers, which indicates that there is still some work to do in the future to further reduce the noise and increase the mode stability for shorter pulse generations. Note that the terahertz spectral spanning is also a vital factor that should be taken into account for generating ultrashort pulses.

In summary, we have demonstrated a terahertz frequency comb that exhibits significant self‐mode stabilization owing to the incorporation of a multilayer graphene‐integrated saturable absorption mirror into a terahertz QCL. The intermode beat note linewidth is as narrow as 700 Hz. Nonlinear terahertz *z*‐scan measurements for a 15‐layer graphene sample yield a saturation peak intensity of 7.3 ± 0.12 W cm^−2^ and a saturable absorption coefficient of 0.21 ± 0.01 at 4.2 THz. The on‐chip dual‐comb has been successfully performed to prove the passive comb operation in the terahertz laser. Furthermore, a pulse width of 16 ps has been obtained from a terahertz pump–probe experiment, which directly proves the pulse generation in GiSAM‐QCLs. The possible origins of the enhanced passive frequency comb operation and pulse generation, i.e., saturable absorption and/or dispersion compensation introduced by the graphene sample, are discussed in detail. The highly stabilized passive frequency comb offers a powerful tool that can be used in terahertz metrology applications. For instance, the dual‐comb technique enables fast spectroscopy with Doppler‐limited resolution. The ultrashort terahertz pulses generated from GiSAM‐QCL combs could also find promising uses in studying the carrier dynamics of various materials, such as 2D materials and III‐V semiconductors.

## Experimental Section

*Terahertz QCL*: The terahertz QCL used in this work was based on a hybrid active region structure that combines the bound‐to‐continuum optical transitions and the resonant phonon scattering for depopulation. The layer sequence of one period for the QCL active region with 76 cascade periods, from the injection barrier, is **4.1**/3.8/**1**/23.6/**1**/13.8/**2.1**/11.8/**3.1**/9.6/**3.1**/8.7/**3.1**/7.7/**3.1**/17.2/**3.4**/14.8 nm, where Al _0.25_Ga_0.75_ As layers are listed in bold, GaAs layers in normal font, and the 17.2 nm thick GaAs layer (in underlined font) is doped to 2 × 10^11^ cm^−2^. The entire QCL active region was grown using a molecular beam epitaxy system on a semi‐insulating GaAs substrate. The grown wafer was then processed into single plasmon waveguide laser ridges using traditional GaAs processing technology, such as optical lithography, electron beam evaporation, chemical and dry etching, lift‐off, etc. The ridges were cleaved into laser bars with cavity lengths ranging from 2 to 6 mm and then indium‐soldered onto copper bases (heat sink). Finally, the QCL device was mounted onto a cold finger of a cryostat for electrical and optical measurements.

To measure the CW power of the terahertz QCL, two off‐axis parabolic mirrors were used for collecting and focusing the terahertz light onto a terahertz sensor (Ophir, 3A‐P THz), which was connected to a power meter for reading the CW power value. The power value shown in Figure S1b in the Supporting Information is the measured power without any calibration of light collection efficiency, window transmission, water absorption, or mirror reflection. The emission spectra shown in Figure 3c and Figure S15 in the Supporting Information were measured using a Fourier transform infrared (FTIR) spectrometer (Bruker, v80). To reduce the water absorption as much as possible, the FTIR chamber was pumped down to a pressure of 3 mbar, and the beam path outside the FTIR was purged with dry air. The spectral resolution was 0.1 cm^−1^ (3 GHz).

*Multilayer Graphene Fabrication and GiSAM Integration*: The multilayer graphene samples were fabricated through a layer‐by‐layer transfer technique of monolayer graphene film grown via chemical vapor deposition. Graphene layer growth was carried out in a quartz tube at reduced pressure with a copper foil (thickness of 25 µm, purity of 99.8%) as the catalyst substrate. During the process, the copper foil was heated up to 1000 °C and kept for 1 h under a 10 sccm flow of hydrogen (≈400 mTorr) to reduce the oxide presence, increase the grain size, and ensure a smooth surface for graphene growth. Next, methane gas with a beam flux of 10 sccm was introduced into the chamber for the graphene synthesis, and the gas pressure was maintained at ≈750 mTorr for 30 min. Following that, the grown sheet was cooled to room temperature in an atmosphere of hydrogen. The transfer of the graphene films onto the double‐side‐polished Si mirror (with a diameter of 10 mm) was realized by utilizing the conventional float‐transfer method. A polymethyl methacrylate resist was used as the protective layer, and the iron chloride etchant was used to etch away the copper substrate. The number of graphene layers on the desired substrate was controlled by the number of times the transfer was repeated. It is worth noting that additional loss was introduced because of the interfacial dangling bonds, and graphene film quality degrades as the number of monolayer graphene transferred increases.

The multilayer graphene and Si mirror constructed a nonlinear reflector, GiSAM, which was then coupled to the 6 mm long terahertz QCL using a sample fixture as shown in Figure S1e in the Supporting Information. It has to be clarified that by coupling the GiSAM into the QCL compound cavity, an etalon consisting of the air gap and the air/Si interface was introduced, which might result in loss modulation and mode selection in the QCL. The modulation period was calculated to be ≈360 GHz by following the formula Δν = *c*/2*ln*, where *c* is the speed of light in vacuum, *l* = 410 µm is the length of the air gap (see Figures S4 and S5 in the Supporting Information), and *n* is the refractive index of air. Since the modulation period was much larger than the emission spectral range shown in Figure 3c and Figure S15 in the Supporting Information, the mode selection caused by the etalon effect played a negligible role on the passive comb formation. In the QCL configuration, the air gap was 410 µm wide, which was much larger than the typical gap width in micrometer level normally employed in coupled‐cavity lasers. The etalon effect introduced by such two‐mirror Si could be reasonably neglected. Note that the air gap and the air/Si interface formed a GTI mirror and the dispersion introduced by the latter is clearly elaborated in the main text and the Supporting Information.

*Open‐Aperture z‐Scan*: As shown in Figure S9a in the Supporting Information, the terahertz light emitted from the GiSAM‐QCL was collimated and then focused onto the graphene sample by a pair of off‐axis parabolic mirrors with a reflected focal length of 101.6 mm. The sample was placed on a motorized micrometric stage (Newport, M‐ILS200CCL) moving along the optical axis (*z*). After being transmitted through the graphene sample, the light was collected and focused onto a terahertz power detector using another pair of parabolic mirrors, and the power value was read using a power meter (Ophir, 3A‐P THz). The beam focal spot size (*w*_0_) was measured to be 209 µm, with a Rayleigh length (*z*_R_) of 600 µm. The open‐aperture *z*‐scan was performed at different QCL drive currents (700, 800, and 900 mA) by recording transmittance versus sample position along the *z*‐axis. The step size of the stage was set to 0.4 mm. For each QCL drive current, the measurement of the transmitted terahertz intensity was repeated 10 times and the mean value for fitting was taken. The transmittance of the 0.3 mm thick Si‐substrate was also recorded and fitted with a third‐order polynomial function, which was used as a baseline for data normalization. Details of the fitting procedure can be found in the Supporting Information.

*Intermode Beat Note Measurement*: The intermode beat note spectra were measured electrically using a spectrum analyzer with the assistance of a broadband Bias‐T (Marki, BT2‐0026). To extract the high‐frequency signal from the laser cavity, a microwave transmission line as shown in Figure 1a was used. The transmission line was mounted behind the back facet of the QCL. The connection between the laser bar and the transmission line was achieved using wire bonds. The beat note signal extracted from the transmission line was sent to the AC port of the Bias‐T and then measured using a spectrum analyzer (Agilent Technologies, N9020A). The intermode beat note spectra shown in Figure 2a were measured in “trace average” mode with an average number of 20. The resolution bandwidth used in this measurement was 100 kHz. The beat note spectra shown in Figure 2b were measured in the “max‐hold” mode. Once the “max‐hold” function was switched on, the spectrum analyzer recorded the spectral maxima in a store mode, enabling to see the frequency drift over a limited time. Here, the time duration was 3 min for “max‐hold” measurements.

*On‐Chip Dual‐Comb*: The on‐chip dual‐comb device that was investigated here consisted of two terahertz QCL combs, one of which acted as a local oscillator (LO) comb (Comb1) and the other one was a sample comb (Comb 2) (see Figure 3a). The two QCL combs with same ridge dimensions (150 µm × 6 mm) were on a same chip processed from the same wafer. Due to the strong nonlinearity of QCL chips, the intermode beat note and dual‐comb spectra could be measured electrically using the LO comb (Comb1) as a fast detector and finally recorded using a spectrum analyzer (Rohde & Schwarz FSW) with an assistance of a low noise amplifier (30 dB gain) as shown in the inset of Figure 3a. To avoid optical injection locking, the two combs were spatially separated by 1.6 mm.

To perform the dual‐comb measurement, the intermode beat note signals of both QCL combs were monitored. It was essential to first observe two single narrow beat note frequencies to ensure that both QCLs were working as frequency combs. To successfully obtain the dual‐comb spectra, the repetition rates (intermode beat note frequencies) of both combs should be slightly different. Otherwise, no dual‐comb spectrum can be observed. Once two single narrow intermode beat notes as shown in Figure 3a are obtained, the down‐converted dual‐comb lines below the intermode beat note frequency (≈6.2 GHz) can be searched.

*Terahertz Pump–Probe*: The terahertz pump–probe experiment was carried out by exploiting the nonlinearity (saturable absorption) of the graphene sample. As the graphene film can be saturated by the terahertz light (see Figure 1d), the terahertz pulses emitted from the GiSAM‐QCL can then be sampled by employing a pump–probe experiment as shown in Figure 4a. The pulse sampling was made possible by adding a delay line into the optical path of the probe beam which changed the arriving time of the probe beam at the graphene sample. To detect the transmitted power intensity of the probe beam, a sensitive terahertz camera (NEC, IR/V‐T0831C) was employed rather than an average power detector. In the experiment, the beam pattern at each time delay was recorded. To get the power intensity, the integral of the beam pattern was then calculated. The time evolutions of the beam pattern and power intensity are depicted in Video S2 in the Supporting Information. Note that the pump–probe experiment is extremely sensitive to the optical alignment, QCL electrical pumping condition, heat sink temperature, mechanical vibrations, and all other noises.

## Conflict of Interest

The authors declare no conflict of interest.

## Supporting information

Supplementary

Supplementary

Supplementary
