# Supplementary material for: Graphene‐Coupled Terahertz Semiconductor Lasers for Enhanced Passive Frequency Comb Operation
Source: Adv Sci (Weinh). 2019 Aug 23;6(20):1900460. doi: 10.1002/advs.201900460 (PMC6794721; doi:10.1002/advs.201900460)
Supplement: Supplementary file 1 — Supplementary [file ADVS-6-1900460-s002.pdf]

Copyright WILEY-VCH Verlag GmbH & Co. KGaA, 69469 Weinheim, Germany, 2019.

ADVANCED SCIENCE
Open Access

Supporting Information

for *Adv. Sci.*, DOI: 10.1002/advs.201900460

Graphene-Coupled Terahertz Semiconductor Lasers for Enhanced Passive Frequency Comb Operation

Hua Li, Ming Yan, Wenjian Wan, Tao Zhou, Kang Zhou, Ziping Li, Juncheng Cao,* Qiang Yu, Kai Zhang,* Min Li, Junyi Nan, Boqu He, and Heping Zeng**

Supporting Information

Graphene-coupled terahertz semiconductor lasers for enhanced passive frequency comb operation

Hua Li*, Ming Yan, Wenjian Wan, Tao Zhou, Kang Zhou, Ziping Li, Juncheng Cao*, Qiang Yu, Kai Zhang*, Min Li, Junyi Nan, Boqu He, and Heping Zeng*

(Optional Dedication)

Prof. Hua Li, Dr. Wenjian Wan, Tao Zhou, Kang Zhou, Ziping Li, Prof. Juncheng Cao
Key Laboratory of Terahertz Solid State Technology, Shanghai Institute of Microsystem and Information Technology, Chinese Academy of Sciences, Shanghai, China.
E-mail: hua.li@mail.sim.ac.cn; Email: jccao@mail.sim.ac.cn

Prof. Hua Li, Kang Zhou, Ziping Li, Prof. Juncheng Cao
Center of Materials Science and Optoelectronics Engineering, University of Chinese Academy of Sciences, Beijing 100049, China.

Prof. Ming Yan, Junyi Nan, Boqu He, Prof. Heping Zeng
State Key Laboratory of Precision Spectroscopy, East China Normal University, Shanghai 200062, China.
Email: hpzeng@phy.ecnu.edu.cn

Qiang Yu, Prof. Kai Zhang
i-Lab, Suzhou Institute of Nano-Tech and Nano-Bionics, Chinese Academy of Science, 398 Ruoshui road, Jiangsu 215123, China.
Email: kzhang2015@sinano.ac.cn

Min Li, Prof. Heping Zeng
School of Optical Electrical and Computer Engineering, University of Shanghai for Science and Technology, Shanghai 200093, China.

Keywords: terahertz laser, frequency comb, on-chip dual-comb, pulse generation

1 Terahertz QCL and the compound cavity

The terahertz laser is based on a hybrid design that employs bound-to-continuum transitions for terahertz-frequency photon emission and resonant-phonon for fast depopulation of the lower laser state.[1] Figure S1a shows the calculated band structure of the QCL active region by solving the Schrödinger equation at an electric field of 6 kV/cm. The bound-to-continuum transitions between the upper (thick red curves) and lower (thick blue curves) laser states are for generating terahertz photons. To achieve the population inversion between the laser states, the electrons in the continuum band are then depopulated by fast longitudinal optical phonon scatterings. The photon and phonon emissions are schematically indicated by the arrows in Figure S1a. Figure S1b displays the measured light-current-voltage ($L-I-V$) characteristics of the bare laser working in continuous wave (CW) mode at 15 K. The power is measured using a pair of parabolic mirrors for light collection and focus. The power value shown in Figure S1b is the detected power without consideration of the light collection efficiency, water absorption loss in the beam path, polyethylene-window transmission or mirror reflectivity. The measured maximum power is approximately 2 mW per

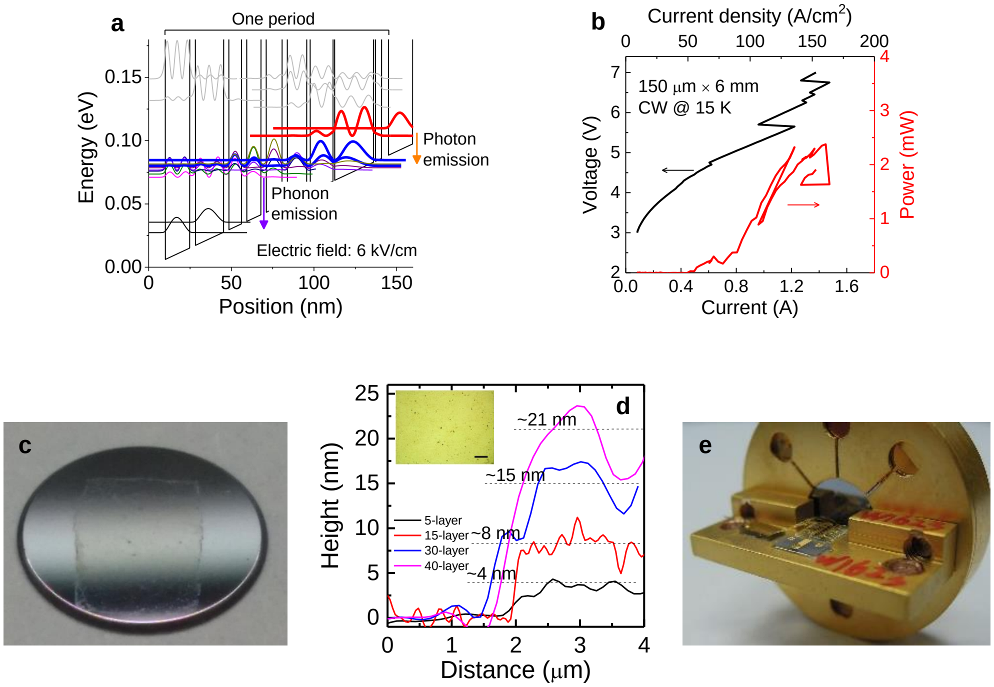

Figure S 1: Terahertz QCL and graphene integration. a) Calculated conduction band structure of the terahertz QCL under an electric field of 6 kV/cm. b) $L-I-V$ characteristics of the 6-mm-long terahertz QCL used in the experiment. The laser operates in CW mode at 15 K. c) Optical image of the 15-layer graphene saturable absorber sample on a 0.3-mm-thick silicon mirror (GiSAM). The diameter of the silicon mirror is 10 mm. d) AFM topography of the graphene films. The inset of d) is an optical microscope image of a 15-layer graphene sample. Scalar bar: 100 μm. e) Photo of the GiSAM-QCL.

facet. From Figure S1b, we can see that apart from the decent CW power, the laser is also characterized by a low operation voltage (5 V), ultralow threshold current density J_{th} (50 A/cm^2) and large dynamic range ($J_{\text{th}} \rightarrow 2J_{\text{th}}$).

Figure S1c shows the optical image of the 15-layer graphene on a silicon mirror. It can be seen that the large-size graphene film is transferred onto the silicon mirror and uniformly distributed on the central part of the mirror. The surface quality and thickness of fabricated multilayer graphene samples are evaluated by atomic force microscopy (AFM) shown in Figure S1d. Since water molecules can be trapped during the graphene transfer process, the thickness of the first layer graphene on Si substrate is approximately 1 nm and the following each graphene layer is 0.33 nm thick. By considering these numbers, the measured thicknesses of the fabricated graphene films qualitatively agree well with the nominal values, which indicates that the graphene layer number can be well controlled. The 15-layer graphene sample on silicon mirror is finally coupled with the terahertz QCL cavity using a delicately-designed sample fixture as shown in Figure S1e.

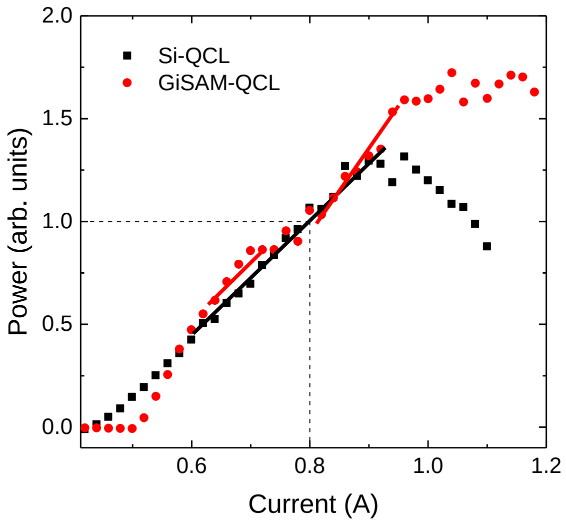

Figure S 2: $L - I$ characteristics of the 6 mm long and 150 μm wide QCL with Si mirror (Si-QCL, squares) and GiSAM (GiSAM-QCL, circles) recorded in constant current mode at 15 K. The power values are normalized to 1 at 800 mA for clear comparison.

Figure S2 shows the comparison of $L - I$ curves taken for the Si-QCL and GiSAM-QCL. It is worth noting that the comparison of absolute power for Si-QCL and GiSAM-QCL doesn't make much sense since the linear losses for both geometries are different. From Figure S2, we can clearly see that the Si-QCL demonstrates almost single slope efficiency in the entire current dynamic range. However, the GiSAM-QCL shows much different behavior. Especially, as the current is larger than 800 mA, the slope efficiency becomes slightly higher as indicated by the red line. Because of this, in Figure S2 the power values are normalized to 1 at 800 mA. The change of slope efficiency of the GiSAM-QCL can be a sign of nonlinearity (saturable absorption) introduced by the graphene sample.

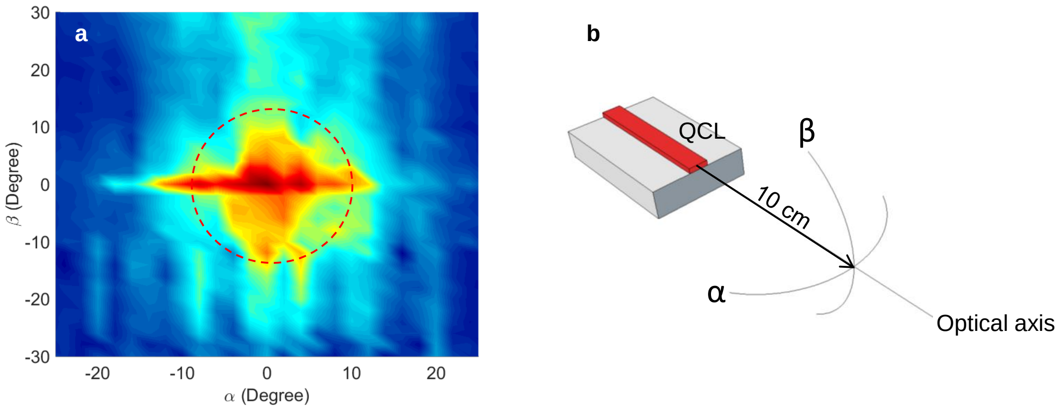

Figure S 3: a) Far-field profile of the 6-mm-long FP-QCL measured at a drive current of 900 mA in pulsed mode. b) Schematic plot of the far-field measurement configuration. α and β denote the horizontal and vertical directions, respectively.

Figure S3a shows the measured far-field pattern of the 6-mm-long FP-QCL without the graphene integrated saturable absorber mirror (GiSAM). The experiment was done by employing a Golay cell terahertz detector rotating on a spherical surface. The QCL was put at the center of the sphere with a radius of 10 cm as shown in Figure S3b. To improve the angular resolution, a pinhole was mounted in front of the terahertz detector. A step of 1° is used for the scanning of the far-field pattern. In Figure S3a, a dashed circle is outlined to show that most of the terahertz light is confined inside this area. In both horizontal and vertical directions (α and β), the divergence angle is measured to be approximately 20°.

Note that the GiSAM is not touching the QCL facet. Therefore, the compound cavity consists of the QCL chip, air gap and GiSAM. For better understanding the dispersion introduced by the external cavity, the air gap length is critical. Figure S4a schematically plots the components of the compound cavity. To prevent the GiSAM from mechanical crack in the mounting process, we use a copper spring as a buffer between the copper heat sink and the GiSAM. The cap with an optical aperture is finally employed to fix the GiSAM to the heat sink. From Figure S4, we can see that the air gap length is equal to the distance from the laser facet to the edge of the heat sink (132 μm, see Figure S4b) plus the thickness of the spring (280 μm, see Figure S4c). Therefore, the total air gap length is approximately 410 μm. The air gap together with the 0.3-mm-thick Si actually forms a Gires-Tournois Interferometer (GTI) mirror. This GTI will introduce additional dispersion besides the material, gain, and waveguide group velocity dispersions in the QCL. In the next section, we will show the effect introduced by the GTI mirror.

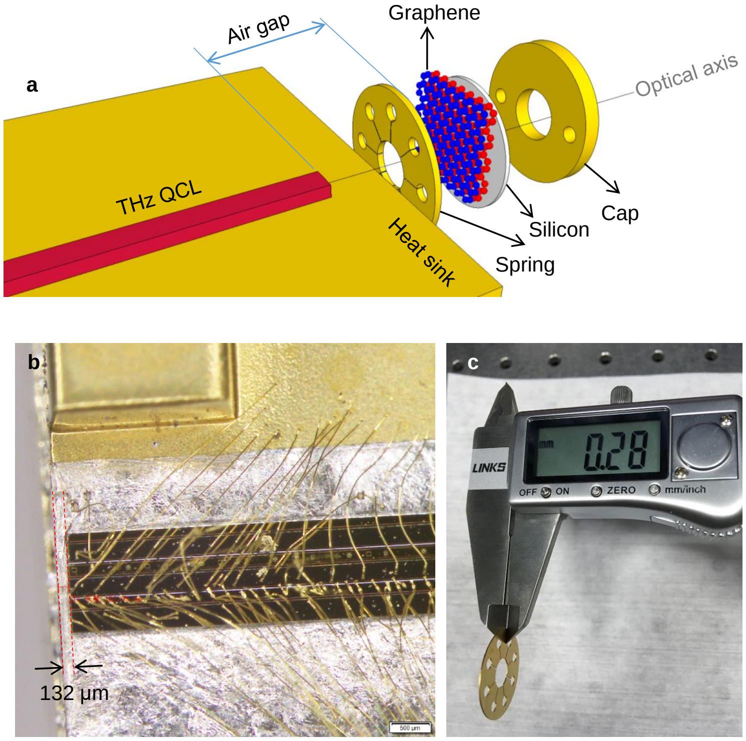

Figure S 4: a) Schematic plot of the components of the compound cavity. b) Optical microscope image of the mounted 6-mm-long QCL chip on a copper heat sink. The distance from the laser facet to the edge of the heat sink is measured to be 132 μm. c) The thickness of the spring measured with a caliper.

2 GTI mirror and dispersions

To understand the effect of the GTI mirror on the dispersion of the QCL, we should first calculate the total disperison of the QCL without GTI mirror, including material, waveguide, and gain group velocity dispersions (GVD) or group delay dispersions (GDD, GDD=GVD$\times L$ and L is the length of the cavity). Thanks to the Kramers-Kronig relations that describe the frequency dependence of wave propagation (refractive index) and attenuation (loss or gain), the group velocity and GVD can be then calculated as a function of frequency to evaluate the dispersions in the QCL.[2–6]

Figure S5a shows the geometry of the QCL and GTI mirror. Note that to make the GTI simulation simpler, the Fabry-Pérot effect of the Si is not taken into account. This approximation is reasonable because in this compound cavity geometry the air gap is far wider than the air gap used in a typical GTI compensator. Therefore, the Si etalon can be neglected. Here the GTI consists of the air gap and the air/Si interface. To calculate the GDD that the GTI introduces, the two reflection coefficients, r_1 and r_2, should be first calculated. These parameters can be obtained by performing the finite element simulation shown in Figure S5b. By launching the terahertz signal

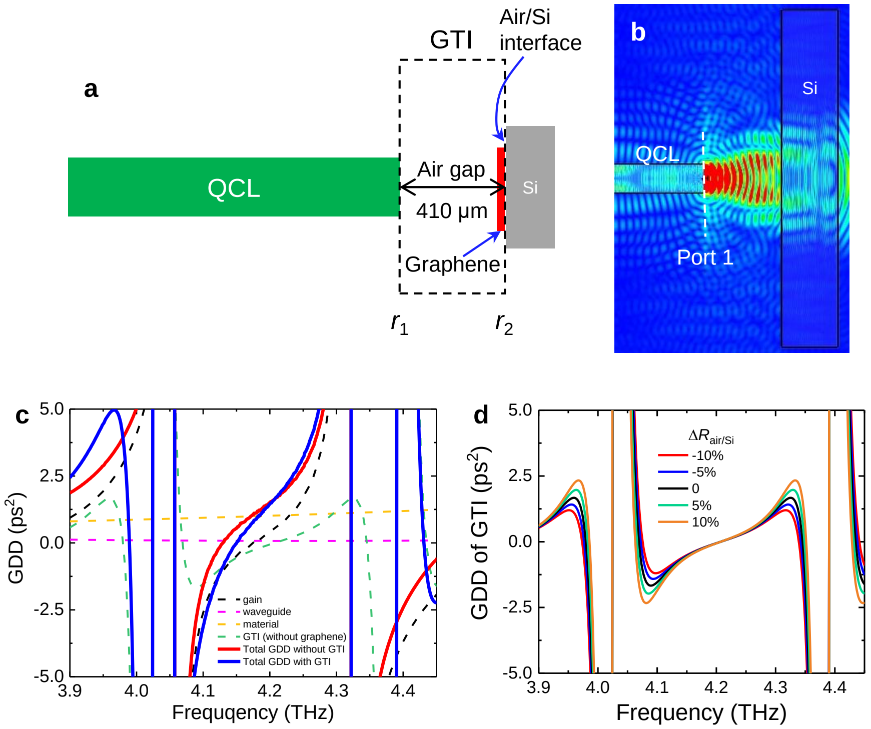

Figure S 5: a) Schematic plot of the geometry of the GTI mirror. b) Finite element simulation of the QCL with the GTI mirror. The terahertz signal is launched at "Port 1" (laser facet) towards the GTI. c) Calculated group delay dispersions (GDD) of the gain, waveguide, material and GTI without graphene. The thick red and blue curves show the total GDD without and with the GTI contribution, respectively. d) GDD of GTI with graphene by considering various changes in reflectivity ($\Delta R_{\text{air/Si}}$).

at "Prot 1", the S_{11} can be calculated and then the reflection coefficients can then by calculated by employing the scattering matrix of a two-mirror cavity. Finally, in Figure S5c we show the simulated dispersions, including the gain, waveguide, material, and GTI GDD. Without the GTI, the total GDD is shown as the red cruve and the main contributions come from the material and gain. As shown in Figure S5c, in the working frequency range of the QCL, the GDD of GTI slowly increases with frequency and from negative to positive. When we compare the total GDD without and with the GTI, we can see that the GTI does compensate the total dispersion as the frequency is below 4.2 THz. While, as the frequency is higher than 4.2 THz, the GTI brings about additional dispersion. Since the calculated GDD of GTI is small between ± 1.25 ps^2, the GTI does not significantly alter the overall dispersion of the laser.

In Figure S5d, the dispersion of GTI with graphene is evaluated for various reflectivity changes ($\Delta R_{\text{air/Si}}$). The GDD of GTI without graphene ($\Delta R_{\text{air/Si}}$=0) is taken from Figure S5c as a reference. It can be seen that the decrease or increase in reflectivity introduced by graphene results in different dispersion compensation behaviours. Specifically, for a -10% reflectivity change in-

troduced by graphene, the total dispersion can be compensated, which can favor the passive comb operation of the GiSAM-QCL. While, if the reflectivity is increased, the total dispersion of the laser will be even worse.

3 Multilayer graphene transmission calculation

To calculate the transmission of the multilayer graphene, we first examine the optical sheet conductivity, $\sigma(\omega)$, of the monolayer graphene, which is related to the thin-film transmission. The real part of the optical conductivity of monolayer graphene can be written as follows:[7]

$$\begin{aligned}\frac{\sigma_{\mathrm{r}}(\omega)}{\sigma_Q} =& \frac{8k_BT}{\pi\hbar}\ln(e^{-E_f/2k_BT}+e^{E_f/2k_BT})\frac{1}{\omega^2\tau+1/\tau} \\ &+\frac{1}{2}\left[\tanh\left(\frac{\hbar\omega+2E_f}{4k_BT}\right)+\tanh\left(\frac{\hbar\omega-2E_f}{4k_BT}\right)\right].\end{aligned} \quad (1)$$

Here ω is the frequency, k_B is the Boltzmann constant, T is the temperature, $\hbar$ is the reduced Planck constant, E_f is the Fermi energy, τ is the momentum scattering time. The first term of Eq. 1 is the Drude-like intraband conductivity, and the second term is the contribution from interband transitions. σ_Q=$\frac{\pi e^2}{2h}$ is the universal quantum conductivity. Once the optical conductivity of mono-

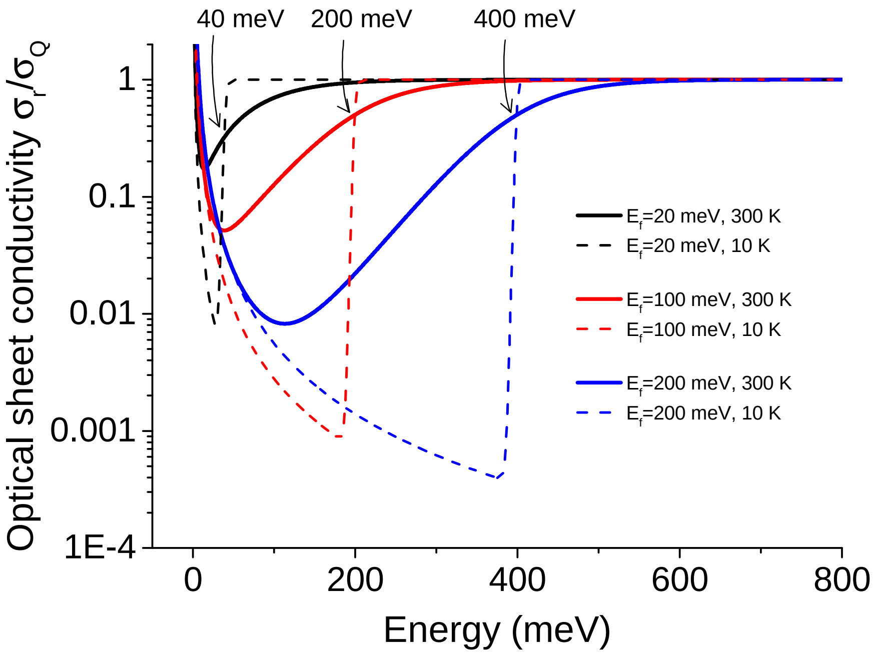

Figure S 6: Calculated conductivity of monolayer graphene. Calculated optical sheet conductivity of monolayer graphene as a function of energy at different temperatures, with solid lines corresponding to a room temperature of 300 K and dashed lines corresponding to a temperature of 10 K. Black plots: E_f= 20 meV; red plots: E_f=100 meV; blue plots: E_f=200 meV.

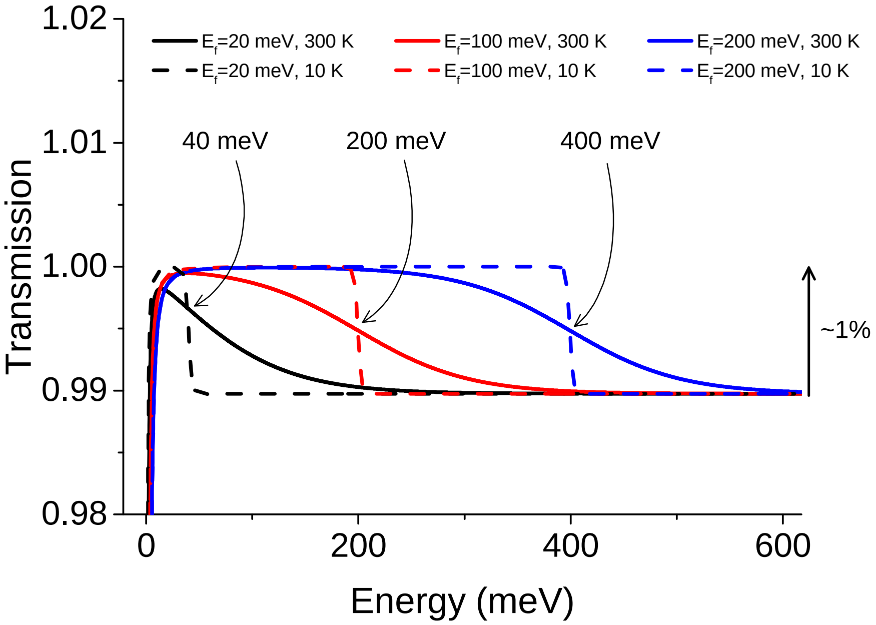

Figure S 7: Transmission of monolayer graphene. Calculated transmission of monolayer graphene using Eq. 2 at different temperatures, with solid lines corresponding to a room temperature of 300 K and dashed lines corresponding to a temperature of 10 K. Black plots: E_f= 20 meV; red plots: E_f=100 meV; blue plots: E_f=200 meV.

layer graphene is known, we can calculate the transmission of N-layer graphene normalized to the substrate transmission using the following relation:[8]

$$T(\omega) = \left| \frac{1}{1 + NZ_0\sigma_{\rm r}/(1+n_S)} \right|^2. \tag{2}$$

Here Z_0=$\frac{1}{\varepsilon_0 c}$ is the vacuum impedance, with ε_0 the vacuum permittivity and c the speed of light, N is the number of graphene layers, and n_S the refractive index of the substrate. Note that in Eq. 2, the contribution of the imaginary part of the conductivity is neglected.

The Fermi energy (E_f) of the fabricated graphene is a critical ingredient that enables us to correctly fit the measured transmission of the graphene samples. As shown in Figure S6, the calculated optical sheet conductivity of the monolayer graphene depends strongly on the Fermi energy E_f, that is, the conductivity exhibits different behaviour below and above the optical energy level of $2E_f$. The difference in conductivity is more pronounced at a low temperature of 10 K. In Figure S6, we show the calculated results for different E_f values and, for each case, we can clearly see there is a sudden change in conductivity at $2E_f$. The underlying mechanisms have already been clarified in the literatures.[9, 10] When the optical energy exceeds $2E_f$, the conductivity (or absorption) of graphene originats from the interband transitions; in the vicinity of $2E_f$, both interband and intraband transitions play a role; and if the photon energy is smaller than $2E_f$,

the interband transition is no longer possible, and only the intraband process contributes to the absorption.

In Figure S7, we show the calculated transmission of monolayer graphene. As shown in Figure S6, the transmission is accordingly dependent on the Fermi energy of the graphene material. One thing that should be noted is that from the calculation, the change in transmission in the vicinity of $2E_f$ is very small, approximately 1%, and it is therefore difficult to clearly observe the difference experimentally. However, the results shown in Figure S7 provide a way to determine the Fermi energy of graphene.

4 Fermi level of the fabricated graphene

As discussed in the previous section, we can measure the transmission of the graphene film to derive the Fermi energy of the fabricated sample. Here, we employ a Fourier transform infrared

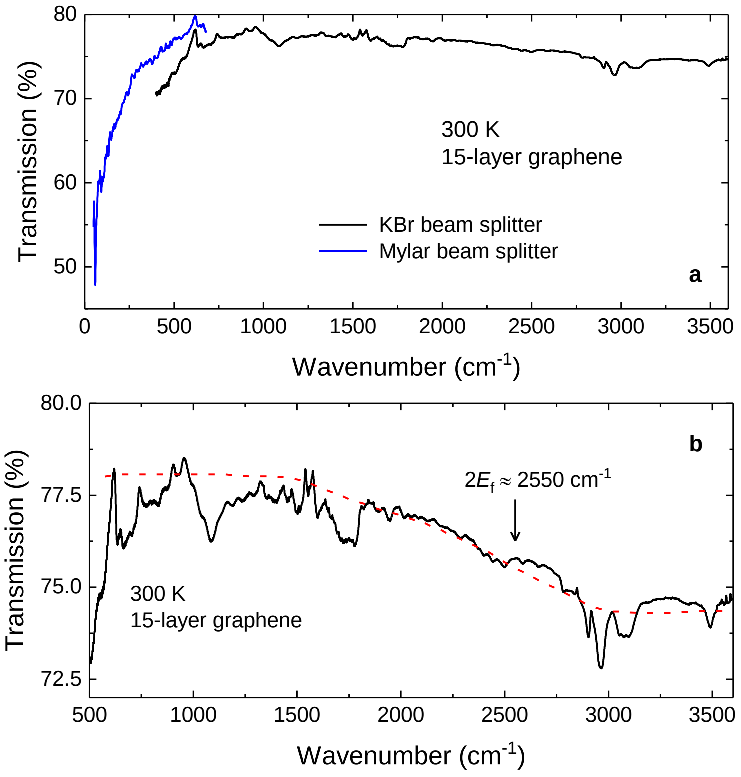

Figure S 8: Transmission of the 15-layer graphene sample. a) Measured transmission of the 15-layer graphene sample in the mid-infrared wavelength range (black line) and terahertz frequency range (blue line) at room temperature. The black line is measured with a KBr beam splitter, while the red line is taken with a 50-μm-thick Mylar beam splitter. b) Zoom-in view of the transmission in the mid-infrared range shown in a). The dashed line provides a guide for the eyes.

(FTIR) spectrometer to perform the transmission measurements in the mid-infrared wavelength range.[11] The transmission spectrum is obtained by taking the ratio of the spectrum derived using the 15-layer graphene sample with Si mirror to that derived from a 0.3-mm thick Si mirror. For the spectral measurement, the FTIR chamber is under vacuum.

As seen in Figure S8a, the transmission spectra are measured for the 15-layer graphene in both mid-infrared (black line) and terahertz (blue) frequency ranges. Note that the terahertz spectrum (blue line) is taken from Figure 1c in the main paper. The discrepancy in transmission around 500 cm^{-1} is due to the fact that the two spectra were taken using different beam splitters. Although the transmission values are not exactly same for the two spectra, the trend with frequency is similar. As we already mentioned, the change in transmission around $2E_f$ is very small, to see this small change we plot the zoom-in view of the transmission spectrum in Figure S8b where we clearly observe a drop in transmission with increasing frequency. The mid-point of the decrease in transmission is measured to be 2550 cm^{-1} (see the dashed line). We know that the Fermi level of the graphene sample is the key parameter for understanding the optical absorption (dynamic conductivity) process. As seen in Figure S8b, we experimentally observe the transmission change at the photon energy of $2E_f$ (2550 cm^{-1}, $\sim$316 meV), therefore, the value of E_f is derived to be approximately 158 meV below the Dirac point. In the terahertz range, the photon energy is far smaller than twice the value of the Fermi energy of the graphene sample, thus terahertz-light-induced absorption can only be due to the intraband transition process.[9] By employing the Drude intraband model,[7] we perform a theoretical fitting (dashed lines in Figure 1c of main paper) using fitting parameters, specifically, momentum scattering time τ and E_f (Eq. 1).

5 z-scan and fitting

The nonlinear behaviour of the 15-layer graphene sample is studied using the z-scan technique. The experimental setup of z-scan is shown in Figure S9a. The terahertz source is the 6-mm-long QCL with a GiSAM, and the transmitted power is measured with a terahertz power meter. Using this approach, we vary the diameter of the terahertz beam waist to gradually tune the terahertz power density by moving the sample along the optical axis (see the Methods for details). If terahertz saturable absorption occurs, we are able to see an increase in the transmission at z_0 where the terahertz power density reaches a maximum. Figure S9b plots the waist diameter of the terahertz beam (measured using a terahertz camera) as a function of z. The red curve shows a fit to the measured data, yielding a beam waist diameter of 209 μm at focal point z_0 and a Rayleigh length of 600 μm. The beam pattern at z_0 is shown in Figure S9c, and we can clearly see that a Gaussian beam is obtained at z_0. We note that when z is far (approximately 1 mm) away from z_0, the beam profile becomes distorted, and non-Gaussian beams are obtained (see Supplementary Video 1). Therefore in Figure S9b, the beam waist diameters for z positions far from z_0 are estimated from the measured 2D beam profiles.

For the purpose of fitting the measured z-scan traces, the widely used two-level saturable

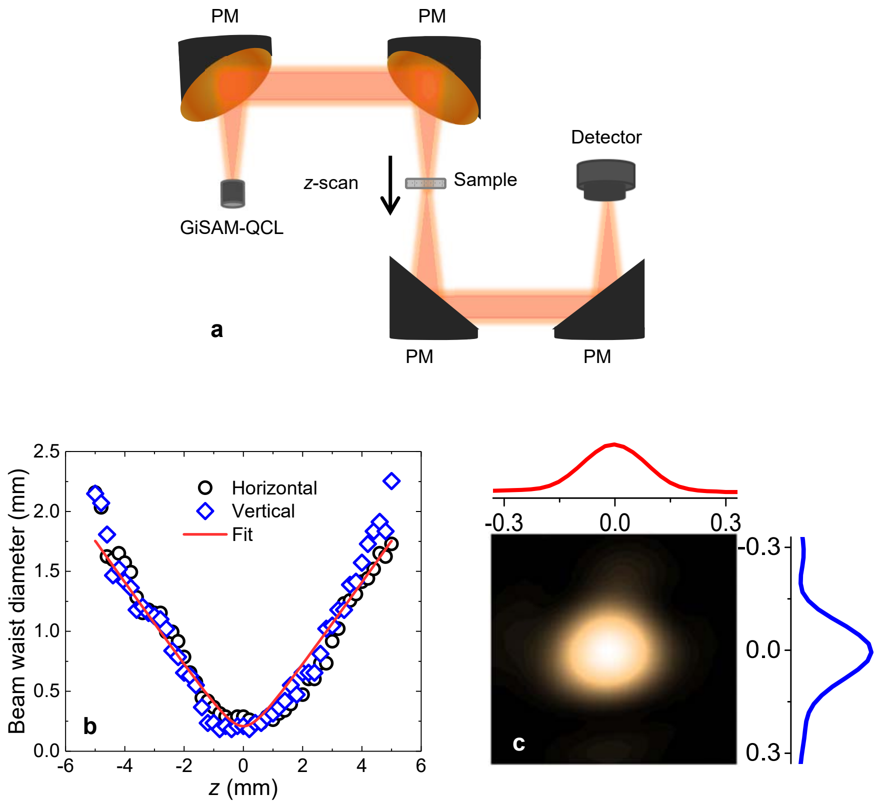

Figure S 9: a) z-scan experimental setup. PM: parabolic mirror. b) Beam waist diameter of the terahertz light measured in horizontal (circles) and vertical (diamonds) directions as a function of position along the optical axis z. The red line is a fit using the fitting function $w(z) = w_0\sqrt{1 + (\frac{z}{z_R})^2}$, where w_0 is the beam waist diameter at position $z = 0$ (z_0) and z_R is the Rayleigh length. c) 2D beam profile of the QCL with a GiSAM measured at z_0 using a terahertz imager. The QCL is pumped at a drive current of 900 mA. On the top and right sides, the horizontal and vertical line profiles of the beam are given. The area shown in the figure is 0.66×0.66 mm^2.

absorber model[12–14] is adapted to yield the dependence of the sample absorption, $\alpha(I)$, on the radiation intensity, I, as:

$$\alpha(I) = \frac{\alpha_{\mathrm{S}}}{1 + I/I_{\mathrm{s}}} + \alpha_{\mathrm{NS}}. \tag{3}$$

Here α_{S} and α_{NS} are the saturable and nonsaturable absorption coefficients, respectively. The saturation intensity, I_{S}, is defined as the light intensity required for half of the saturable absorption. Here, the light intensity (I) as a function of the sample position along the z-axis can be written as:

$$I = \frac{I_0}{1 + z^2/z_{\mathrm{R}}^2}. \tag{4}$$

To extract the absorption coefficients (α_{S} and α_{NS}) and the saturation intensity (I), the normalized

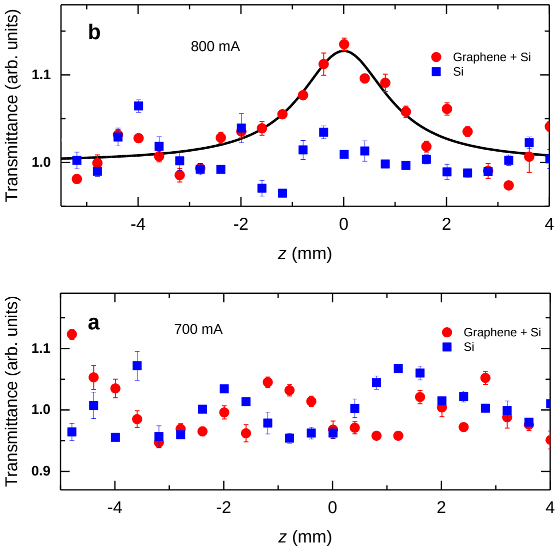

Figure S 10: Transmission of a 15-layer graphene sample using the z-scan technique. Normalized transmission of a 15-layer graphene sample measured at GiSAM-QCL drive current of 700 mA a) and 800 mA b).

z-scan trace of the graphene sample is fitted by the following equation:[15, 16]

$$T(z) = 1 + \frac{\alpha_S}{1 - (\alpha_S + \alpha_{NS})}\left(1 - \frac{1 + z^2/z_R^2}{1 + z^2/z_R^2 + I_0/I_S}\right). \quad (5)$$

Here the two fixed parameters, I_0 and z_R, are obtained from the measrued beam diameter shown in Figure S9.

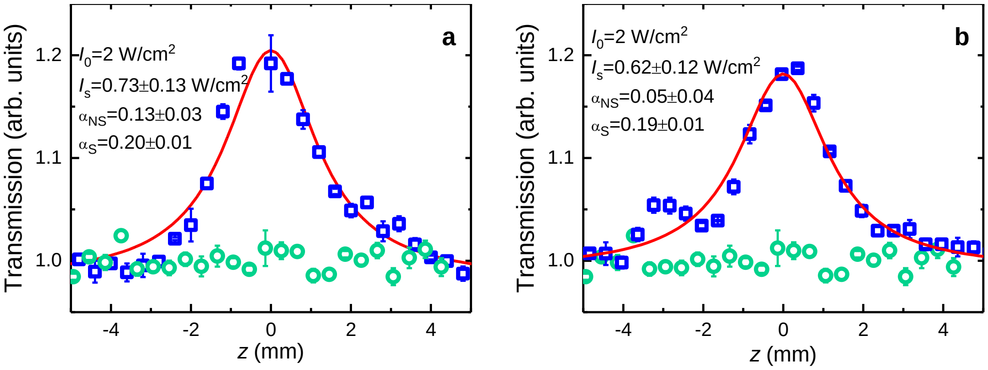

Figure S 11: a) and b) are two z-scan traces measured at different spots of the 15-layer graphene sample as the GiSAM-QCL is operated at 900 mA. The green circles show the transmission of Si which are taken from Figure 1d. The scatters are measured results and the red curves are fits to the experimental data.

As a supplement to Figure 1d of the main paper, we also show here the z-scan traces mea-

sured at different QCL drive currents. In Figure S10, the normalized transmission of the 15-layer graphene is plotted as a function of z for QCL drive currents of 700 (a) and 800 mA (b). It can be seen that at a drive current of 700 mA, because the laser power measured at 390 μW is not sufficient to saturate the graphene sample, saturation behaviour cannot be observed. As the drive current is increased to 800 mA (laser power is 623 μW), saturable absorption arises, as shown in Figure S10b. Using the fitting technique described above, the parameters of the saturation absorption for an 800-mA current are found to be α_N=0.09±0.04, α_S=0.15±0.03, and I_S=0.91±0.42 W/cm^2.

To further prove that the saturable absorption measured at 900 mA shown in Figure 1d is not due to non-uniformity of the fabricated graphene sample, in Figure S11 we show another two measurements at different spots of the sample. As expected, the measured z-scan traces shown in Figure S11 are almost identical to the one shown in Figure 1d. This indicates that the graphene sample is uniform on the surface and the saturable absorption of the graphene sample is confirmed.

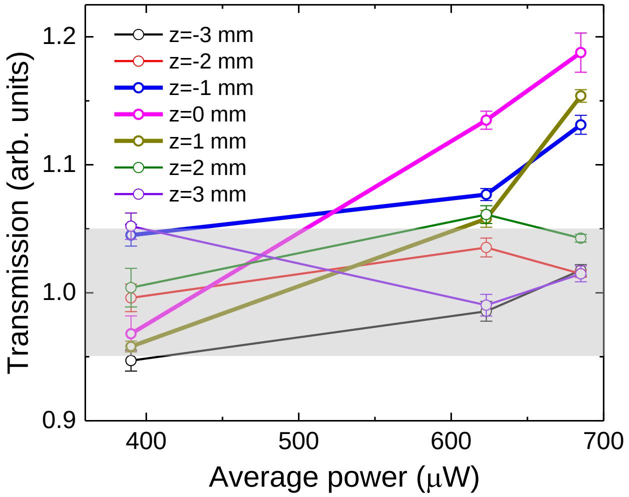

Figure S 12: Power-dependence of transmission for different z positions. The GiSAM-QCL is employed as a THz radiation source. The grey area shows the transmission of Si obtained from Figure S10.

Figure S12 shows the normalized transmission of the graphene sample as a function of power of the GiSAM-QCL for various z positions. It can be seen that with increasing the average power, the increase of transmission is clearly observed for z=-1, 0, and 1 mm. The results shown in Figure S12 also confirm the nonlinearity of the fabricated graphene sample.

6 Short-cavity terahertz QCL integrated with multilayer graphene

We already showed that the GiSAM significantly improved the mode stability of a 6-mm long terahertz QCL. In this section, we investigate whether the GiSAM can exert similar effects on

short-cavity terahertz QCLs. The short-cavity laser ridge is cleaved from the same processed QCL wafer as the 6-mm long device. Figure S13 shows the measured $L-I-V$ characteristics of a 3-mm long and 200-μm wide QCL in CW mode at 10 K. The laser can produce 2.5 mW of CW power at 10 K, and the threshold current density is slightly higher than that of the 6-mm long laser due to the larger mirror loss. Similar as what observed in Figure S1b, when the current density is higher than 120 A/cm^2, the current oscillation can be clearly observed due to the negative differential resistance. Both the threshold current density and the maximum current density measured from the 3-mm device are very close to the values obtained from the 6-mm laser, which shows that the molecular beam epitaxy growth and laser fabrication process are uniform and reliable.

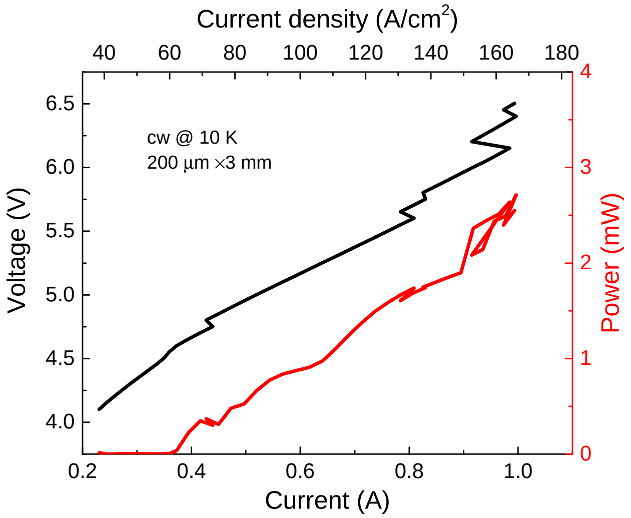

Figure S 13: Device performance of the short-cavity terahertz QCL. The measured CW $L-I-V$ characteristics of a 3-mm long and 200-μm wide terahertz QCL. Data were recorded at 10 K.

In Figure S14, we compare the electrical inter-mode beat note spectra for both the FP-QCL and the GiSAM-QCL. Figure S14a shows that the FP-QCL demonstrates large phase noise. We can see that the beat note spectra are always broad for currents ranging from 450 to 850 mA. At a current of 850 mA, the beat note spectrum spans over 500 MHz. Once the current exceeds 900 mA, the beat note signal disappears, indicating that the laser mode coherence is completely lost. A comparison of Figure S14a and Figure 2 of the main paper reveals that the 3-mm long FP-QCL exhibits much worse frequency stability than the 6-mm FP-QCL. The similar phenomenon was also observed in Ref. [17]. The underlying mechanism is that as the cavity length is increased, the mode spacing decreases. And therefore, the locking introduced by the four-wave mixing becomes much stronger and finally we observe the mode stability improvement in the long cavity lasers.

Figure S14b displays the beat note mapping obtained with the 3-mm long GiSAM-QCL. It is clear that after integrating the GiSAM, the fine structure of the beat note spectral changes. At most currents, the beat note spectra display multiple lines, either broad or narrow. Although single narrow beat note spectra are not obtained, the mode coherence is improved as a result of

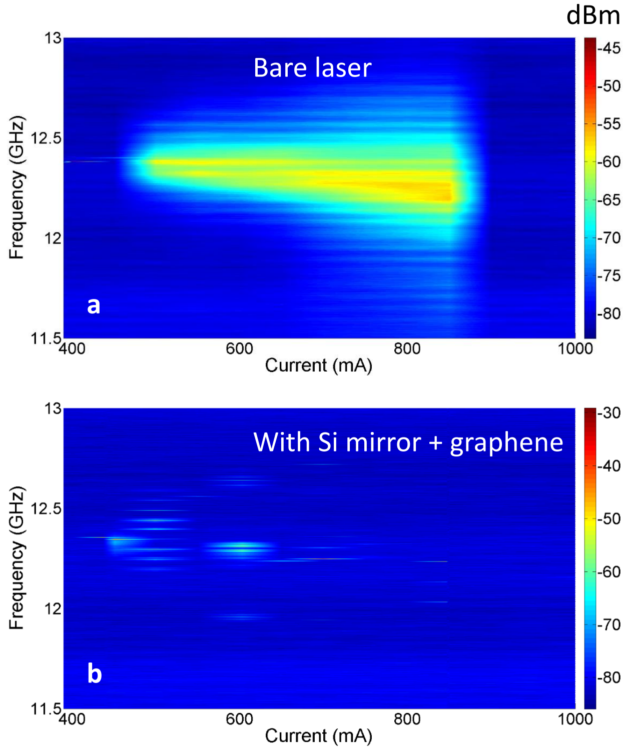

Figure S 14: Electrical inter-mode beat note mapping for the short cavity terahertz QCL. Beat note frequency evolution as a function of drive current for a) the bare QCL and b) the QCL with Si mirror and 15-layer graphene absorber .

incorporating the GiSAM. As for the 3-mm long FP-QCL, as the current is increased above 900 mA, the beat note signal disappears for the GiSAM-QCL.

Our comparison of the short- and long-cavity (3 mm versus 6 mm cavity) lasers leads us to conclude that GiSAM can improve the mode coherence of terahertz QCLs and the frequency stability for comb operation. However, because the short-cavity laser without GiSAM demonstrates a degraded mode coherence than the long-cavity QCL, the graphene absorber has less of an advantageous impact for a short-cavity QCL.

7 Emission and dispersion analysis

Figure S15a shows the terahertz emission spectra of FP-QCL (black line), GiSAM-QCL (blue line), and hybrid QCL (GiSAM-QCL under RF injection, red line) measured at different drive currents using a FTIR with a resolution of 0.1 cm^{-1}. The emission spectra demonstrate the frequency coverage of the terahertz QCL with different configurations. For hybrid QCL, the spectral coverage is greater than 200 MHz at most of drive currents. Figure S15b depicts the number of modes

as a function of current for different QCL configurations. We can see that the mode proliferation is strongly enhanced by RF injection.

Regarding the frequency comb operation, the group-velocity dispersion is critical since it can result in a change in mode spacing with frequency. Limited by the spectral resolution of the state-of-art terahertz spectrometers (in MHz level), the mode spacings of terahertz QCLs cannot be accurately measured. The dispersion can be indirectly characterized roughly by the electrical beat note linewidth shown in Figure 2 of the main paper. The narrower the beat note linewidth, the smaller the dispersion. Here, we use a zero padding technique [5,18] to directly measure the mode spacing of the terahertz spectrum. This involves adding more points in the interferograms for the Fourier transform and then interpolating the terahertz spectrum. The technique can smooth

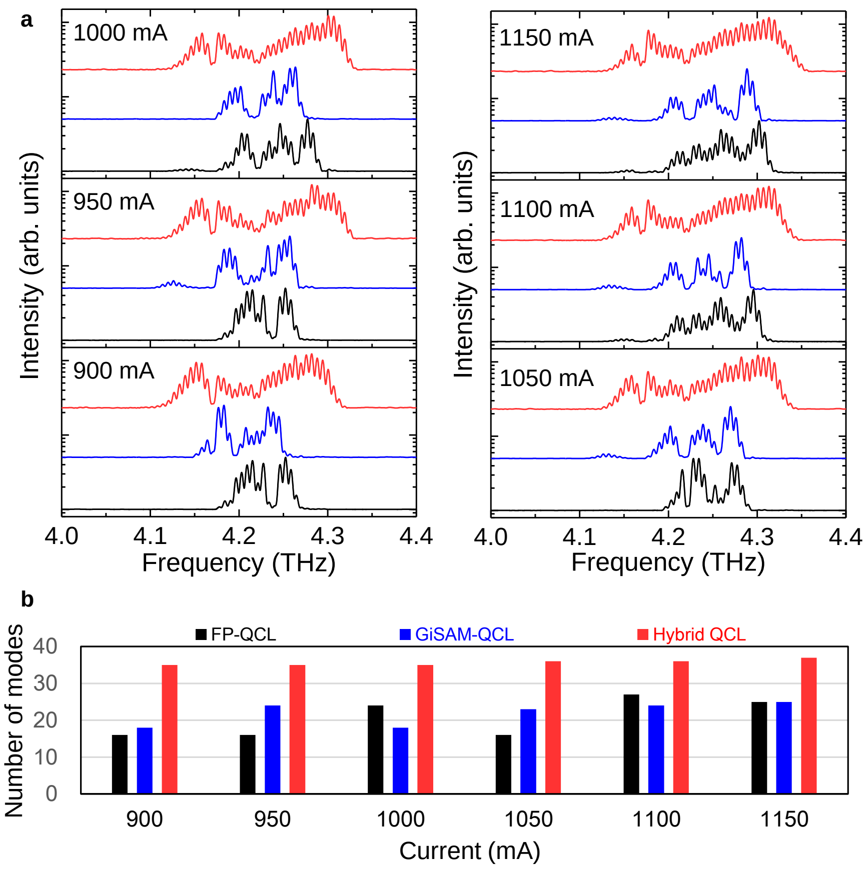

Figure S 15: Terahertz emission spectra. a) Terahertz emission spectra measured at different drive currents for FP-QCL (black line), GiSAM-QCL (blue line), and hybrid QCL (red line). For active RF injection, the frequency of the RF synthesizer is set to be equal to the inter-mode beat note frequency of the free-running GiSAM-QCL. In each panel, offsets are intentionally added to vertically shift the spectra for clarify. b) Number of terahertz longitudinal modes counted within a 20-dB range as a function of drive current for the three different QCL configurations.

the spectrum and help accurately identify the mode peaks.

In Figure S16a and S16b we plot the peak frequency and residuals as a function of mode number for different device geometries at drive currents of 900 and 1000 mA, respectively. The black crosses in Figure S16 are the peak-frequency positions identified from the above-mentioned zero-padding technique, and the green lines show the corresponding linear fits. Due to strong water absorption, some modes are missing, especially for the FP-QCL, and the mode spacing or residuals at those points therefore do not reflect reality. However, such absorption does not affect dispersion evaluation of the overall quality of modes. Normally the small residuals randomly distributed around 0 indicate a small dispersion. In that case, we can see that the GiSAM-QCL demonstrates excellent frequency comb operation with smaller dispersion than the FP-QCL and hybrid QCL. Note that for the hybrid QCL, the trends in residuals indicate that when the spectrum is broadened by the RF injection, the GiSAM cannot well control the dispersion of the laser. Due to the small

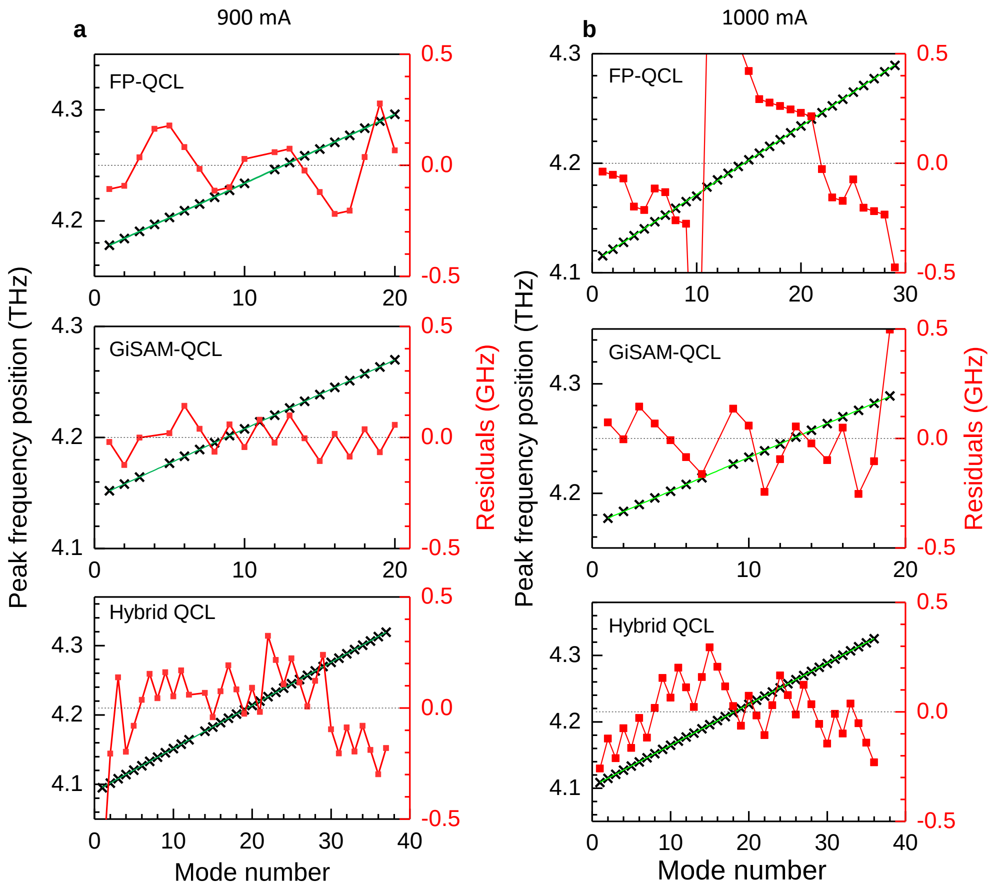

Figure S 16: Dispersion analysis. Measured peak frequency (black crosses, left y-axis) and residuals (red squares, right y-axis) as a function of mode number. The data are recorded at drive currents of 900 mA a) and 1000 mA b). The top, middle, and bottom panels correspond to FP-QCL, GiSAM-QCL, and hybrid QCL, respectively.

dispersion in GiSAM-QCLs, we observe a narrower "max-hold" beat note linewidth down to 30 kHz (see Figure 2 of the main paper) and "single shot" beat note linewidth of 700 Hz (see Figure 3 of the main paper).

8 Dual-comb of FP-QCLs and Si-QCLs

In Figure 2 of the main paper, we show the dual-comb spectrum of GiSAM-QCL combs. For a comparison, in this section we show the dual-comb results obtained from FP-QCLs. The dual-comb device is the same as the one reported in Figure 3 of the main paper. The only difference in the device configuration is that the GiSAM is removed. The measurement setup is exactly same as the one described in the inset of Figure 3a of the main paper.

The inter-mode beat note spectra are shown in Figure S17a measured with a resolution bandwidth (RBW) of 100 kHz. It was measured when the two FP-QCLs are electrically-pumped simultaneously. We can clearly see that two separated beat note frequencies are obtained and the spacing of the two lines is measured to be approximately 22 MHz. The linewidths of the two beat note lines are depicted in Figure S17b. To perform a fair comparison, here we use a same RBW (2

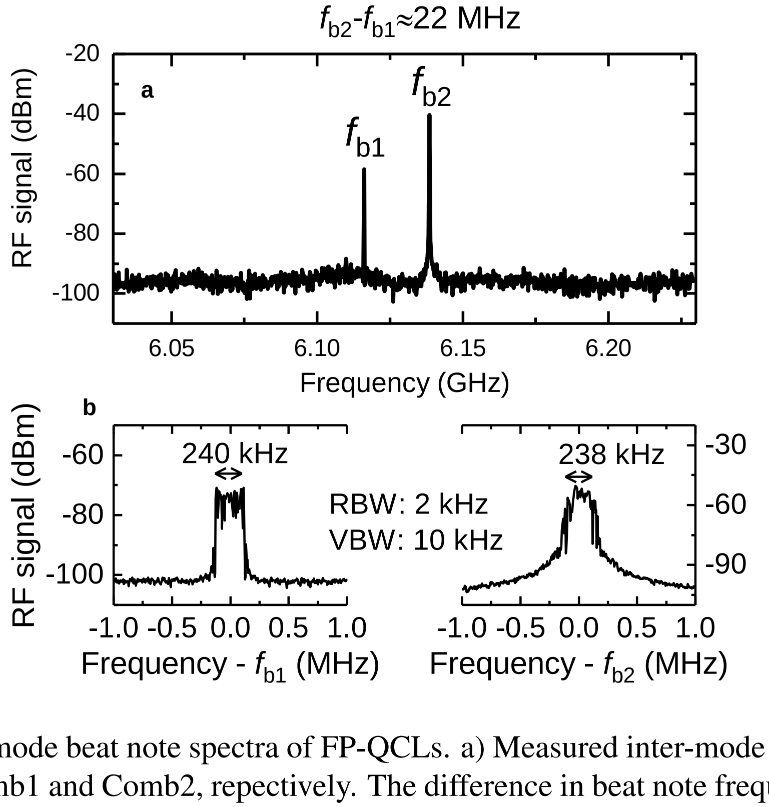

Figure S 17: Inter-mode beat note spectra of FP-QCLs. a) Measured inter-mode beat notes f_{b1} and f_{b2} of FP-QCL Comb1 and Comb2, repectively. The difference in beat note frequency is measured to be approximately 22 MHz. b) Linewidths of f_{b1} (left panel) and f_{b2} (right panel) measured with a RBW of 2 kHz.

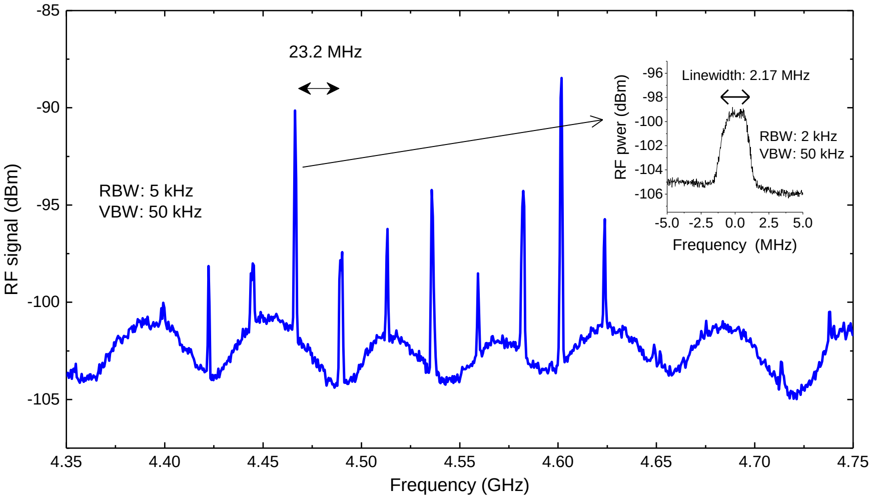

Figure S 18: Dual-comb spectrum measured with a RBW of 5 kHz and a video bandwidth (VBW) of 50 kHz. The line spacing is measured to be 23.2 MHz. The inset shows the typical linewidth of a down-converted dual-comb line.

kHz) parameter for the linewidth measurement. It can be seen that for both beat note lines of FP-QCLs, the linewidths are in a level of 200 kHz which is significantly broader than the linewidths of GiSAM-QCLs (see Figure 3b of the main paper). The comparison of “single shot” linewidths agrees well with the “max-hold” linewidth comparison shown in Figure 2 of the main paper. Both measurements show that the GiSAM significantly increases the frequency stability of QCLs for the frequency comb operation.

In Figure S18, we demonstrate the dual-comb spectrum measured electrically using the FP-QCL Comb1 as a detector. Even without GiSAM, the dual-comb spectrum is also successfully obtained. However, we can notice that the number of mode is less than the dual-comb spectrum obtained from GiSAM-QCL combs. There are 11 visible lines in Figure S18, while in Figure 3d the number of down-converted dual-comb lines is 19. Furthermore, we can clearly see the difference in the linewidth of the dual-comb lines. The typical dual-comb linewidth of the FP-QCLs is measured to be approximately 2.17 MHz as shown in the inset of Figure S18, while the typical linewidth of GiSAM-QCL dual-comb is 720 kHz (see the inset of Figure 3d of the main paper).

Figure S19 plots the terahertz emission spectra of FP-QCL Comb1 (black) and Comb2 (red). The emission spectrum of one FP-QCL comb is recorded when the other FP-QCL is electrically switched off. The inset shows the zoom-in of the spectrum outlined by a dashed rectangle. The frequency difference between the two modes of two combs is measured to be approximately 4.8 GHz which can roughly determine the central frequency of the dual-comb spectrum shown in Figure S18. Because the spectral resolution of the recorded terahertz spectra is low, the two spectra shown in Figure 3c of the main paper for GiSAM-QCLs and Figure S19 for FP-QCLs look identi-

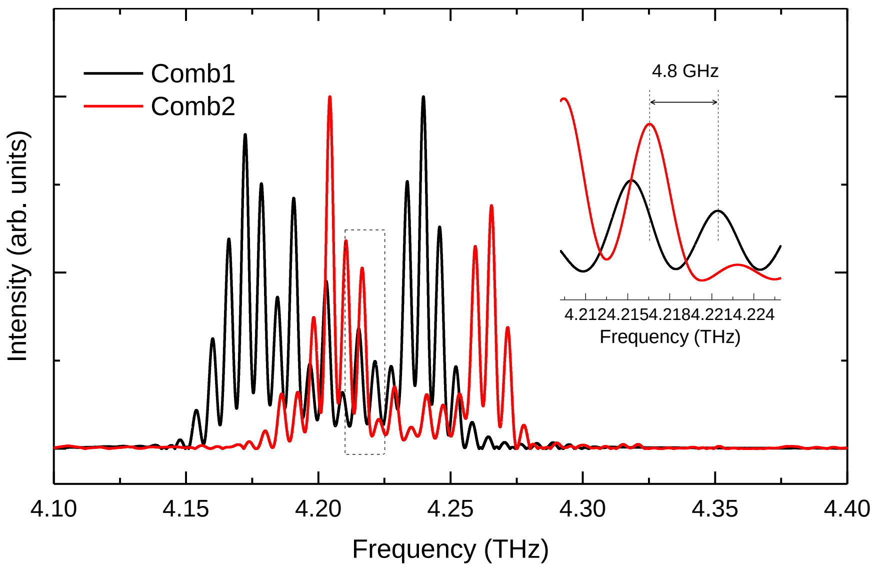

Figure S 19: Terahertz emission spectra of FP-QCL Comb1 (black) and Comb2 (red). The inset shows a zoom-in of the spectrum outlined as a dashed rectangle.

cal, i.e., similar frequency coverage and mode overlap. However, the dual-comb spectra are much different between GiSAM-QCLs and FP-QCLs due to the improved mode coherence of GiSAM-QCLs.

Figure S20 demonstrates the on-chip dual-comb results of Si-QCLs. For the dual-comb experiment of Si-QCLs, the electrical pumping conditions and the heat sink temperature are kept the same as the FP-QCL (see Figures S17-S19) and GiSAM-QCL (Figure 3) dual-comb measurements. Figure S20a shows the emission spectra of Si-QCL Comb1 (black) and Comb2 (red). In both Figures S20a and S19, we can count that 18 terahertz modes of one laser overlap with the other 18 modes of the other laser. However, the dual-comb spectra are quite different. Much worse than the FP-QCLs, as shown in Figure S20b, the Si-QCLs show less dual-comb lines. Even with naked eyes, we can see the lines in Figure S20b are not equally-spaced. The measurement shown in Figure S20 infers that the Si mirror does not always contribute to the comb formation in 6 mm long QCLs. Furthermore, we can conclude that even the inter-mode beat note signals of Si-QCLs are narrow and the emission spectra are strongly overlapped, the frequency comb operation is not obtained in the Si-QCLs.

[1] M. Wienold, L. Schrottke, M. Giehler, R. Hey, W. Anders, H. T. Grahn, *Appl. Phys. Lett.* **2010**, *97*, 071113.

[2] A. Hugi, G. Villares, S. Blaser, H. C. Liu, J. Faist, *Nature* **2012**, *492*, 229.

[3] D. Burghoff, T. Y. Kao, N. R. Han, C. W. I. Chan, X. W. Cai, Y. Yang, D. J. Hayton, J. R. Gao, J. L. Reno, Q. Hu, *Nat. Photonics* **2014**, *8*, 462.

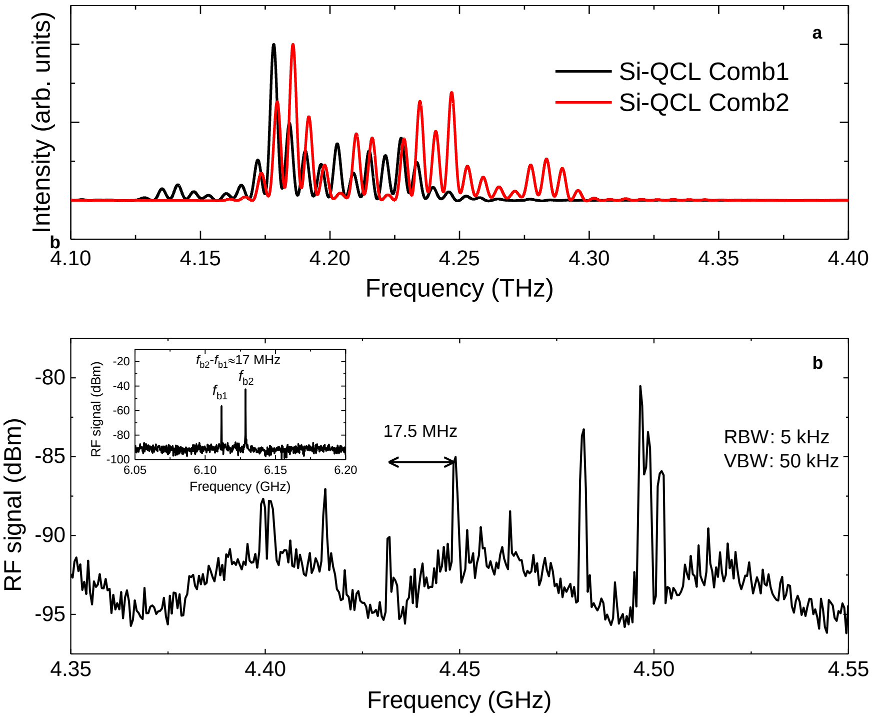

Figure S 20: a) Terahertz emission spectra of Si-QCL Comb1 (black) and Comb2 (red). b) Down-converted dual-comb spectrum of the Si-QCLs. Inset: Inter-mode beat note spectrum of the two Si-QCLs. f_{b1} and f_{b2} denote the beat note frequencies of Si-QCL Comb1 and Comb2, respectively.

[4] G. Villares, J. Faist, *Opt. Express* **2015**, *23*, 1651.

[5] M. Rosch, G. Scalari, M. Beck, J. Faist, *Nat. Photonics* **2015**, *9*, 42.

[6] F. H. Wang, H. Nong, T. Fobbe, V. Pistore, S. Houver, S. Markmann, N. Jukam, M. Amanti, C. Sirtori, S. Moumdji, R. Colombelli, L. H. Li, E. Linfield, G. Davies, J. Mangeney, J. Tignon, S. Dhillon, *Laser & Photonics Reviews* **2017**, *11*, 1700013.

[7] H. Choi, F. Borondics, D. A. Siegel, S. Y. Zhou, M. C. Martin, A. Lanzara, R. A. Kaindl, *Appl. Phys. Lett.* **2009**, *94*, 172102.

[8] P. A. George, J. Strait, J. Dawlaty, S. Shivaraman, M. Chandrashekhar, F. Rana, M. G. Spencer, *Nano Lett.* **2008**, *8*, 4248.

[9] S. Winnerl, M. Orlita, P. Plochocka, P. Kossacki, M. Potemski, T. Winzer, E. Malic, A. Knorr, M. Sprinkle, C. Berger, W. A. de Heer, H. Schneider, M. Helm, *Phys. Rev. Lett.* **2011**, *107*, 237401.

[10] L. Ren, Q. Zhang, J. Yao, Z. Z. Sun, R. Kaneko, Z. Yan, S. Nanot, Z. Jin, I. Kawayama, M. Tonouchi, J. M. Tour, J. Kono, *Nano Lett.* **2012**, *12*, 3711.

[11] K. Zhang, F. L. Yap, K. Li, C. T. Ng, L. J. Li, K. P. Loh, *Adv. Funct. Mater.* **2014**, *24*, 731.

[12] E. Garmire, Ieee J Sel Top Quant 2000, 6, 1094.

[13] Q. L. Bao, H. Zhang, Y. Wang, Z. H. Ni, Y. L. Yan, Z. X. Shen, K. P. Loh, D. Y. Tang, *Adv. Funct. Mater.* **2009**, *19*, 3077.

[14] F. Bianco, V. Miseikis, D. Convertino, J. H. Xu, F. Castellano, H. E. Beere, D. A. Ritchie, M. S. Vitiello, A. Tredicucci, C. Coletti, *Opt. Express* **2015**, *23*, 11632.

[15] Z. W. Zheng, C. J. Zhao, S. B. Lu, Y. Chen, Y. Li, H. Zhang, S. C. Wen, *Opt. Express* **2012**, *20*, 23201.

[16] V. Bianchi, T. Carey, L. Viti, L. H. Li, E. H. Linfield, A. G. Davies, A. Tredicucci, D. Yoon, P. G. Karagiannidis, L. Lombardi, F. Tomarchio, A. C. Ferrari, F. Torrisi, M. S. Vitiello, *Nat. Commun.* **2017**, *8*, 15763.

[17] W. J. Wan, H. Li, T. Zhou, J. C. Cao, *Sci. Rep.* **2017**, *7*, 44109.

[18] P. R. Griffiths, J. A. De Haseth, *Fourier Transform infrared spectrometry*, John Wiley & Sons, **2007**.
